# Differential regulation of expression of the protein kinases DYRK1A and DYRK1B in cancer cells

**DOI:** 10.1038/s41598-024-74190-1

**Published:** 2024-10-13

**Authors:** Vincent Andreas Vorwerk, Gerrit Wilms, Aaron Babendreyer, Walter Becker

**Affiliations:** 1 Institute of Pharmacology and Toxicology, RWTH Aachen University, Wendlingweg 2, 52074 Aachen, Germany; 2 Institute of Molecular Pharmacology, RWTH Aachen University, 52074 Aachen, Germany

**Keywords:** Protein kinase, Kinase inhibitors, XMU-MP-1, Off-target effect, Cell density, AURK, Cancer, Cell biology

## Abstract

The protein kinases DYRK1A and DYRK1B are pivotal regulators of cell cycle progression by promoting cell cycle exit into quiescence. DYRK1B appears to play a more important role in cancer cell quiescence than DYRK1A, as evidenced by its overexpression or copy number variations in human tumour samples. Nonetheless, the stimuli driving DYRK1B upregulation and the potential divergence in expression patterns between DYRK1A and DYRK1B remain largely elusive. In the present study, we scrutinized the regulatory pathways modulating DYRK1B expression relative to DYRK1A in PANC-1 and A549 cancer cell lines across varying conditions. Serum deprivation, pharmacological mTOR inhibition and increased cell density resulted in the differential upregulation of DYRK1B compared to DYRK1A. We then aimed to assess the role of protein kinases MST1 and MST2, which are key transmitters of cell density dependent effects. Unexpectedly, exposure to the MST1/2 inhibitor XMU-MP-1 resulted in increased DYRK1B levels in A549 cells. Further investigation into the off-target effects of XMU-MP-1 unveiled the inhibition of Aurora kinases (AURKA and AURKB) as a potential causative factor. Consistently, AURK inhibitors VX-680 (tozasertib), MLN8237 (alisertib), AZD1152-HQPA (barasertib) resulted in the upregulation of DYRK1B expression in A549 cells. In summary, our findings indicate that the expression of DYRK1A and DYRK1B is differentially regulated in cancer cells and reveal that the kinase inhibitor XMU-MP-1 increases DYRK1B expression likely through off target inhibition of Aurora kinases.

**Supplementary Information:**

The online version contains supplementary material available at 10.1038/s41598-024-74190-1.

## Introduction

Cancer cell quiescence is a common mechanism for cancer cells to survive chemotherapeutic drug treatment and for disease relapse after initial remission. DYRK1 kinases are involved in cell cycle control by stabilizing p27, destabilizing cyclin D1, activating the DREAM complex and thereby promoting cell cycle exit and maintaining cells in a reversible G0 state called quiescence^1–7^.

While DYRK1A and DYRK1B share similar functions in cell cycle regulation, analysis of differential gene expression using the TCGA database revealed that DYRK1B, but not DYRK1A, was upregulated in many human cancer types^8^. Consistent with these findings, numerous pancreatic cancer cell lines such as PANC-1 cells are characterized by increased copy numbers of a 660 kb subregion within the 19q13 chromosome region that includes the *DYRK1B* gene. *DYRK1B* is the only gene in this subregion with a prosurvival function^9^. Moreover, elevated copy numbers of 19q13 correlate with higher pathological stage and higher histological grade of primary pancreatic ductal adenocarcinomas^10^. However, DYRK1B levels are also increased in many tumours without corresponding copy number changes of 19q13^8^. For example, DYRK1B was found to be overexpressed in about 90% of tumour specimens from patients with non-small-cell lung cancer (NSCLC)^11^, compared to its expression in adjacent lung tissue^12,13^. Genetic knockdown of DYRK1B increased the susceptibility of A549 and other NSCLC cells to the cytotoxic effect of cisplatin^11^. This chemosensitizing effect is not restricted to lung cancer cells but extends to various cancer types and cell lines^14^. Moreover, a recent study implicated DYRK1B in conferring radiation resistance^15^. In fact, DYRK1B has been identified as a regulator of DNA damage repair^16^.

Considering the function of DYRK1B in chemoresistance, it is important to understand the mechanism of DYRK1B upregulation, particularly in cancer cells that lack genetic amplification of *DYRK1B*. Like all kinases of the DYRK family, DYRK1B undergoes activation by autophosphorylation and maintains constitutive activity^17,18^. Consequently, the cellular activity of DYRK1B is presumed to primarily depend on its expression level.

DYRK1B levels are increased following growth factor depletion by serum starvation. This effect has originally been observed in U9 colon carcinoma cells^13^ but occurs in many other cancer cell lines including A549^11^ and PANC-1^19^. Pharmacological inhibition of mechanistic target of rapamycin (mTOR), a protein kinase that acts as a key mediator of several growth factors, mimics the effect of serum starvation in PANC-1 cells and other pancreatic carcinoma cells^19^. Similarly, inhibition of the MAPK/ERK pathway by MEK inhibitors upregulated DYRK1B levels in A375^20^ and MelJuso melanoma cell lines^21^. In contrast to mTOR inhibition, AKT and PI3K inhibitors failed to induce DYRK1B expression to the same extent^19^. Notably, the upregulation of DYRK1B does not appear to be an obligatory consequence of G_1_/G_0_ arrest, because treatment with the CDK4/6 inhibitor PD-0332991 (also known as Palbociclib) arrested cells in G_1_/G_0_ phase without affecting DYRK1B levels^21^. Overall, the current evidence regarding the regulation of DYRK1B expression presents several unresolved questions, specifically regarding the diverging expression patterns of DYRK1B and DYRK1A among various human cancers.

In this study, we employed A549 and PANC-1 cancer cell lines to explore the expression of DYRK1B and DYRK1A in response to serum deprivation and increased cell density. Our data demonstrate that DYRK1B, but not DYRK1A, exhibits upregulation on both protein and mRNA levels in response to serum starvation and increased cell density. Through a serendipitous discovery, we also observed upregulation of DYRK1B following treatment with the kinase inhibitor XMU-MP-1, which most likely occurred through off-target inhibition of Aurora kinases.

## Results

### Upregulation of DYRK1B by serum starvation and inhibition of mTORC activity

**Fig. 1:**
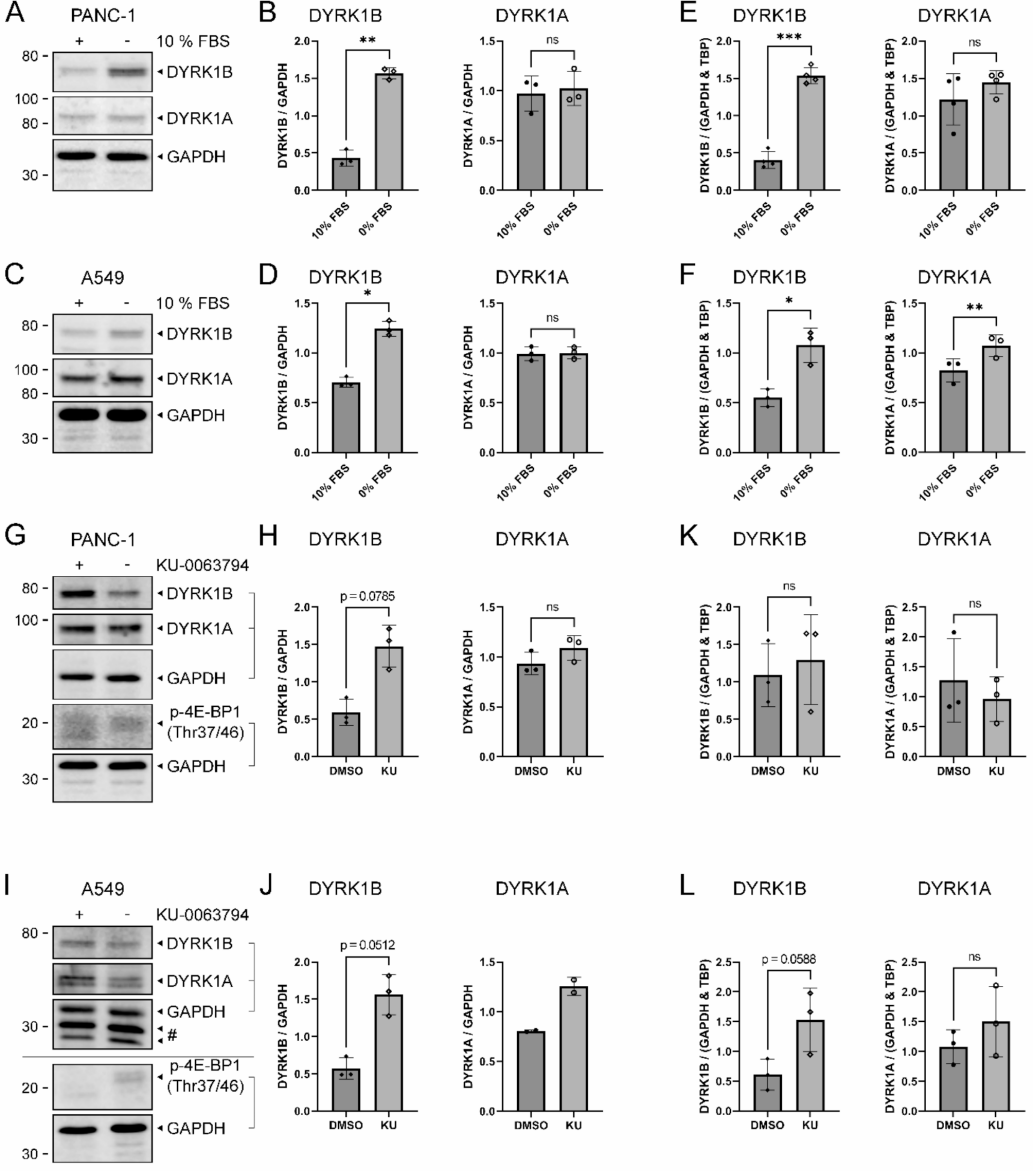
Upregulation of DYRK1B by serum starvation and mTORC inhibition. PANC-1 and A549 cells were cultivated in cell culture medium with (+) or without (−) 10% FBS for 24 h prior to lysis. (**A-D)** Representative Western blots and densitometric analysis; (**E-F**) qPCR analysis. PANC-1 and A549 cells were treated with 1 µM KU-0063749 (+) or the identical amount of DMSO (−) for 24 h prior to lysis. (**G-J**) Representative Western blots and densitometric analysis. Parallel gels with 15% acrylamide were run to detect phosphorylated 4E-BP1 as a control for mTOR inhibition. # marks unspecific bands. The horizontal line in panel I indicates that the blots for DYRK1 and p4EBP-1 originated from different replicas of the same experiment (**K-L**) qPCR analysis. All graphs show means ± SD of *n* = 3 independent experiments except *n* = 4 in panel (**E**) and *n* = 2 for DYRK1A in panel (**J**). Statistical analysis of all data shown in this figure by two-sided paired t-test (* *p* < 0.05; ** *p* < 0.01; *** *p* < 0.001, **** *p* < 0.0001, individual value if *p* < 0.1, ns *p* > 0.1).

Cultivation of PANC-1 (Fig. 1A and B) and A549 cells (Fig. 1C and D) in the absence of fetal bovine serum (FBS) for 24 h caused an increase of DYRK1B protein levels in comparison to control cells grown with 10% FBS. In contrast, DYRK1A levels remained unaltered under serum starvation. qPCR analysis showed that mRNA levels (Fig. 1E and F) were similar to protein expression patterns, except that *DYRK1A* mRNA levels were slightly elevated in serum starved A549 cells.

We used the highly specific mTORC1/2 inhibitor KU-0063794^22^ to pharmacologically simulate serum starvation. In both PANC-1 (Fig. 1G and H) and A549 cells (Fig. 1I and J), inhibition of mTORC1/2 caused elevated levels of DYRK1B protein in each of the replicate experiments, although statistical significance was not reached. *DYRK1B* mRNA levels showed a similar pattern of upregulation in A549 cells (Fig. 1L) but not in PANC-1 cells (Fig. 1K), while *DYRK1A* mRNA levels did not change under KU-0063794 treatment.

## DYRK1B expression in response to long term treatment

**Fig. 2:**
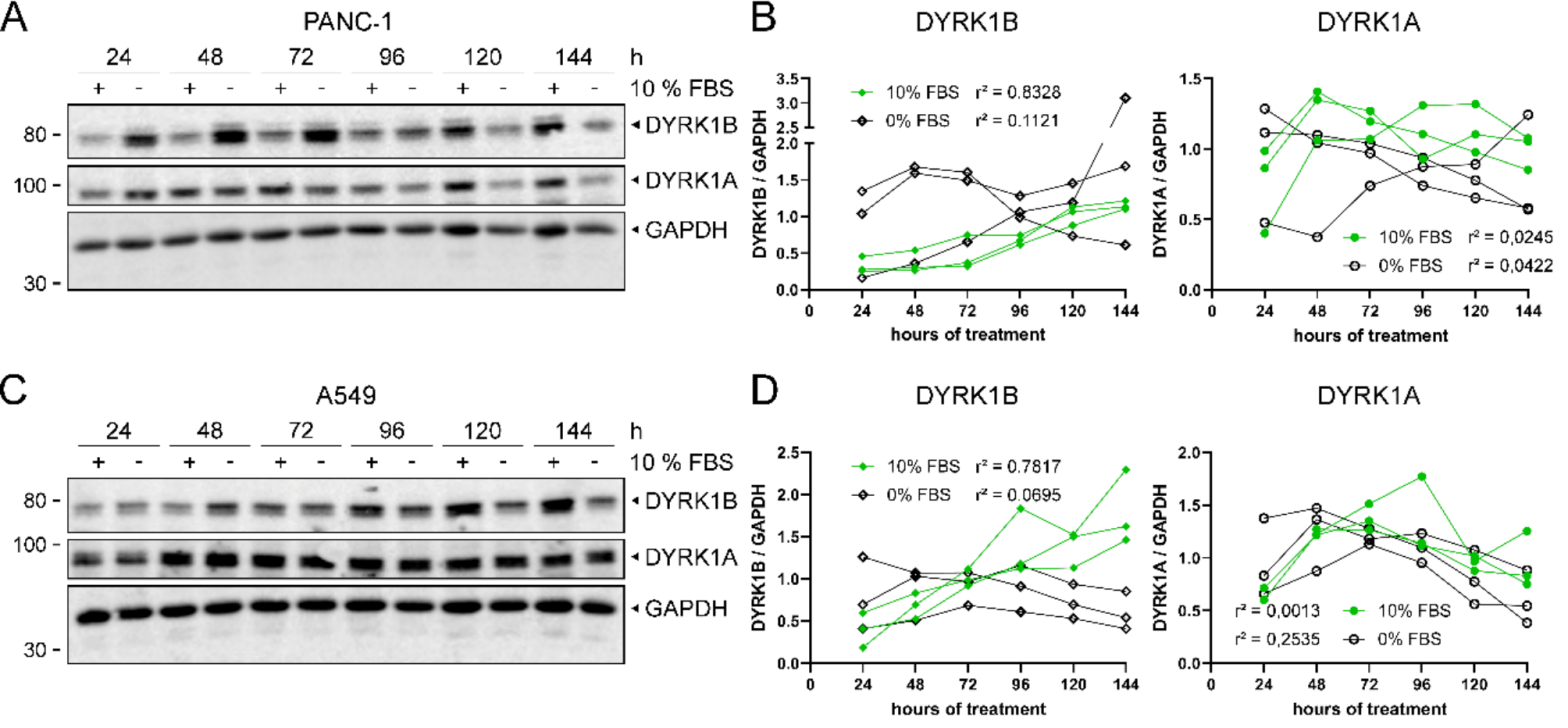
DYRK1B expression in response to long term treatment. PANC-1 and A549 cells were cultivated in cell medium with (+) or without (−) 10% FBS for 24–144 h prior to lysis. Media were changed daily. Representative Western blots and densitometric analysis are shown (PANC-1, **A** and **B**, A549, **C** and **D**). The graphs show individual data points (*n* = 3). Separate quantification of each replicate can be seen in Fig. S2. Statistical analysis by linear regression model (multiple r^2^ as indicated in the figure).

To characterize the time course of DYRK1B upregulation in response to serum deprivation, PANC-1 and A549 cells were cultivated in cell culture media with or without 10% FBS for 24–144 h (Fig. 2A-D). Cell media were replenished daily to prevent mTOR inhibition by depletion of glucose or other nutrients^23^. Consistent with Fig. 1, elevated DYRK1B expression was observed after 24 h of serum starvation in 2 of the 3 replicate experiments in PANC-1 cells and in all replicates of A549 cells. Interestingly, prolonged cultivation in serum-free medium did not further increase DYRK1B levels in correlation to treatment time in a linear model (PANC-1: r^2^ = 0.1121; A549: r^2^ = 0.0695). In contrast, a time dependent increase of DYRK1B protein levels was observed in the control cells treated with 10% FBS (PANC-1: r^2^ = 0.8328; A549: r^2^ = 0.7817). Notably, long term cultivation of A549 cells in the presence of serum resulted in even higher DYRK1B protein levels than serum depletion after treatment for 144 h. DYRK1A protein level did not correlate with treatment time either in the presence or absence of FBS in a linear model. The triplicate band reflects three splice variants of DYRK1B in PANC-1 cells (see Fig. S3 for further details)^24^, while the largest variant is not detectable in A549 cells. Throughout this study, the quantitative evaluation of Western blot results reflects the combined signal intensity of all DYRK1B splicing variants.

We attempted to use reporter gene assays to analyse the effect of serum starvation on DYRK1B promoter activity. The results of these assays remained inconclusive, possibly because relevant enhancer elements were absent in our reporter gene constructs (Figs. S4-S5).

## DYRK1B expression is regulated by cell density

The time course experiment revealed the progressive elevation of DYRK1B levels under conditions where serum was continuously available. We hypothesized that DYRK1B expression was upregulated in response to increasing cell-cell contacts as cells were growing to confluence.

**Fig. 3:**
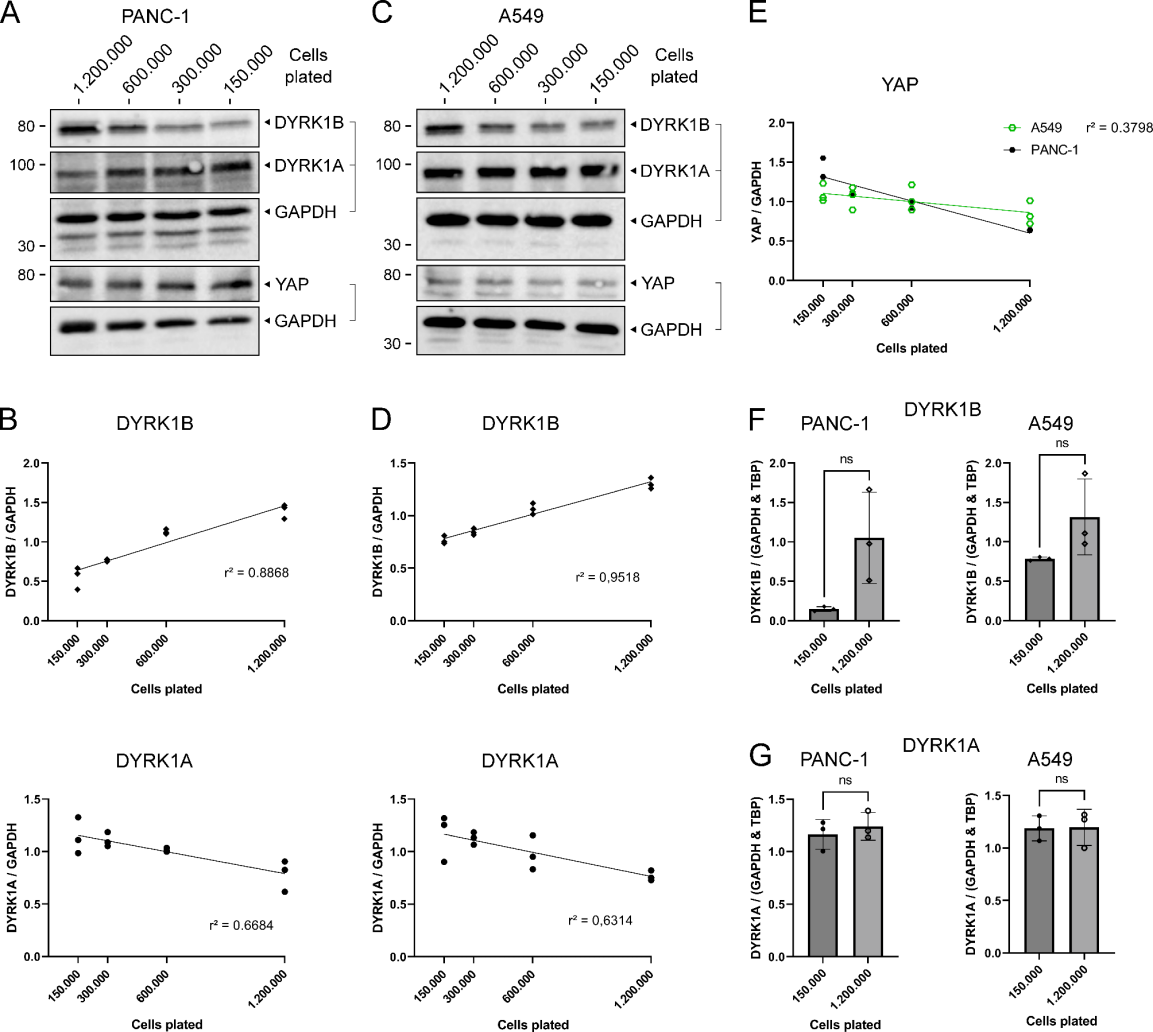
DYRK1B expression is regulated by cell-density. A549 cells were grown in different densities as indicated by the number of cells plated onto each well of a 6 well-plate. Cells were cultivated for 48 h before lysis, and media were changed one day after plating. (**A-E**) Representative Western blots and densitometric analysis of PANC-1 (**A**, **B**) and A549 cells (**C**, **D**). Data are shown as individual data points (*n* = 3 except *n* = 2 for YAP in PANC-1). Statistical analysis by linear regression model (multiple r^2^ as indicated in the figure). (**F**,** G**) qPCR analysis. Data are shown as mean ± SD (*n* = 3). Statistical analysis by two-sided paired t-test (* *p* < 0.05; ** *p* < 0.01; *** *p* < 0.001, **** *p* < 0.0001, individual value if *p* < 0.1, ns *p* > 0.1).

To test this hypothesis, cells were seeded onto 6-well plates at different densities and grown for 48 h. Both PANC-1 and A549 cells showed a strongly density dependent increase of DYRK1B protein levels (PANC-1: r^2^ = 0.8868; A549: r^2^ = 0.9518) (Fig. 3A-D). DYRK1A protein levels were weakly affected and exhibited a moderate inverse correlation with cell density. The transcription factor YAP was used as a control for the cell density effect because abundance of YAP is known to be negatively regulated by cell-cell contacts^25^. Slightly reduced levels of YAP were observed in confluent PANC-1 cells but not in A549 cells (Fig. 3E). Thus, DYRK1B levels appeared to be more strongly affected by cell density than YAP in A549 cells.

Next, we analysed density dependent regulation of DYRK1B expression by qPCR. In all replicate experiments with both cell lines, DYRK1B mRNA levels were higher in high-density grown cells than in the low-density control, although the difference failed to reach statistical significance (Fig. 3F, G). DYRK1A-mRNA levels were not affected in cells grown in different densities. Taken together, these data provide evidence that DYRK1B expression is regulated by cell density.

## DYRK1B is upregulated by treatment with MST1/2 inhibitor XMU-MP-1

**Fig. 4:**
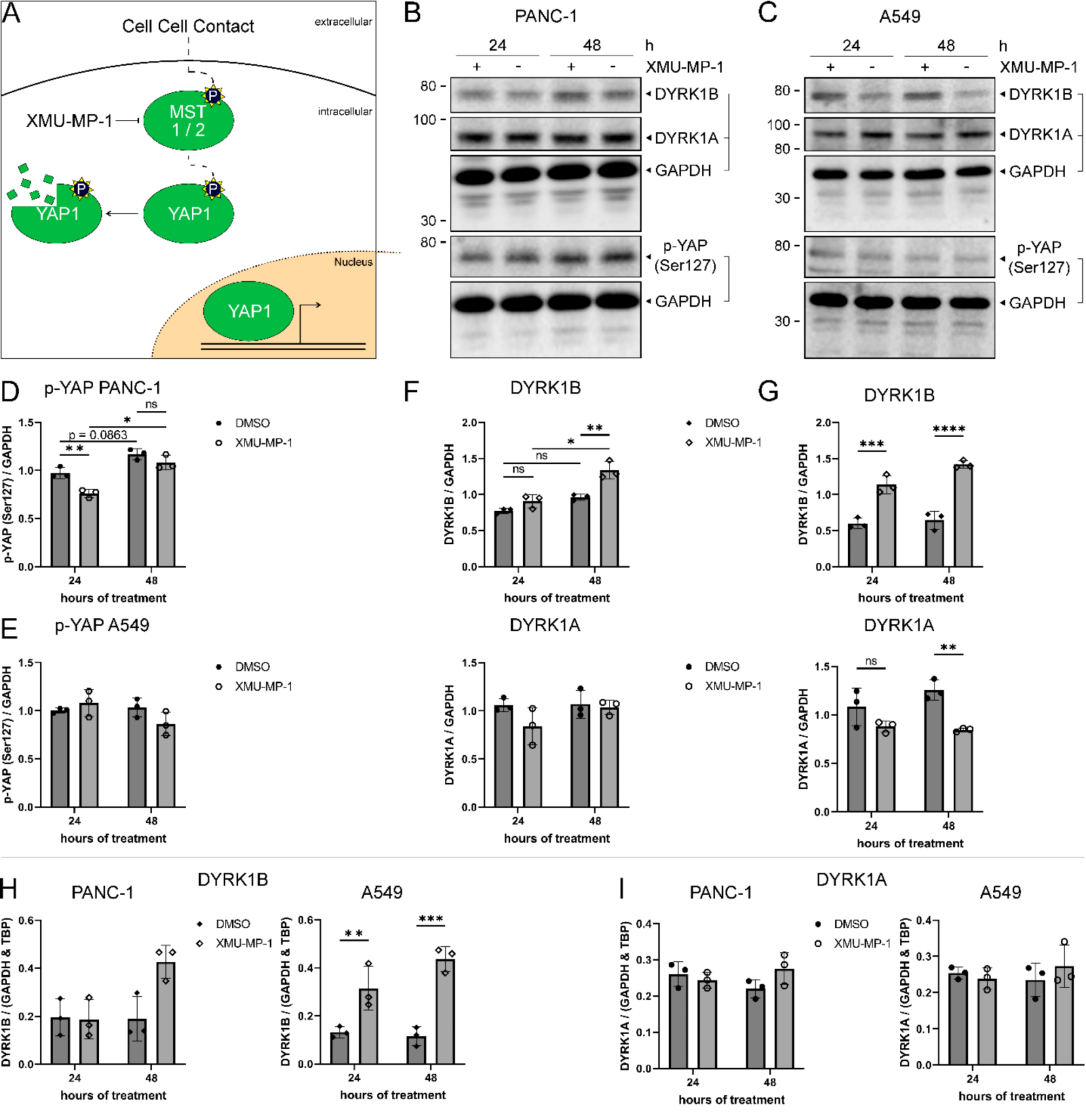
DYRK1B expression is upregulated by the MST1/2 inhibitor XMU-MP-1. (**A**) Simplified scheme illustrating the function of MST1/2 in the Hippo pathway. (**B-G**) Representative Western blots and densitometric analysis of PANC-1 (**B**,** D**,**F**) and A549 (**C**,** E**,**G**) cells treated with 3 µM XMU-MP-1 (+) or DMSO (−) for 24–48 h. Media were changed daily. (**H-I**) qPCR analysis of experiments with conditions identical to (**B-G**). All data are shown as mean ± SD (*n* = 3). Statistical analysis by Two-way repeated measurements ANOVA with Bonferroni multiple testing correction as a post-hoc-test (* *p* < 0.05; ** *p* < 0.01; *** *p* < 0.001, **** *p* < 0.0001, individual value if *p* < 0.1, ns *p* > 0.1).

Next, we asked whether DYRK1B might be a downstream target of pathways involved in contact inhibition. The MST/Hippo pathway plays a critical role in the control of cell proliferation in response to cell-cell contacts. In mammalian cells, this signalling cascade comprises the protein kinases MST1 or MST2, which activate the protein kinases LATS1 or LATS2, which in turn phosphorylate the transcription factor YAP, causing its inactivation and degradation (Fig. 4A)^26^. Therefore, we used the small molecule MST1/2 inhibitor, XMU-MP-1^27^, to interrogate the contribution of this pathway.

The inhibition of MST1/2 by XMU-MP-1 treatment was expected to suppress upregulation of DYRK1B levels under conditions of high cell density. Cells seeded at low density, conditions under which the Hippo pathway is less active, should not be affected by XMU-MP-1 treatment. Contrary to our expectations, an initial experiment showed that XMU-MP-1 treatment did not prevent the increase of DYRK1B levels at high cell densities but rather resulted in an upregulation of DYRK1B in the cells plated at low density (Fig. S6A).

To corroborate this observation, A549 and PANC-1 cells were plated at low density (150.000 cells) and treated with 3 µM XMU-MP-1 for 24–48 h (Fig. 4B-G). These conditions were identified as optimal in dose response curve and time course experiments (Fig. S6B, C). XMU-MP-1 treatment of A549 cells for 24–48 h resulted in an increase of DYRK1B levels and reduced DYRK1A expression after 48 h. Interestingly, the effect of XMU-MP-1 on DYRK1B was much weaker in PANC-1 cells (Fig. 4B, F) than in A549 cells. Phosphorylation of YAP at Ser127 was analysed as a marker of Hippo pathway inhibition. However, phosphorylation of YAP remained unaffected by treatment with XMU-MP-1 in A549 cells or was weakly affected in PANC-1 cells (Fig. 4D, E). Finally, qPCR analysis showed that XMU-MP-1 enhanced the expression of DYRK1B in A549 cells on the level of mRNA abundance (Fig. 4H-I).

Taken together, these results suggest that the XMU-MP-1 treatment upregulates DYRK1B expression in A549 cells independent of MST1/2 inhibition. Thus, no conclusion can be drawn regarding the role of the Hippo-YAP1 pathway in the regulation of DYRK1B expression.

**Fig. 5:**
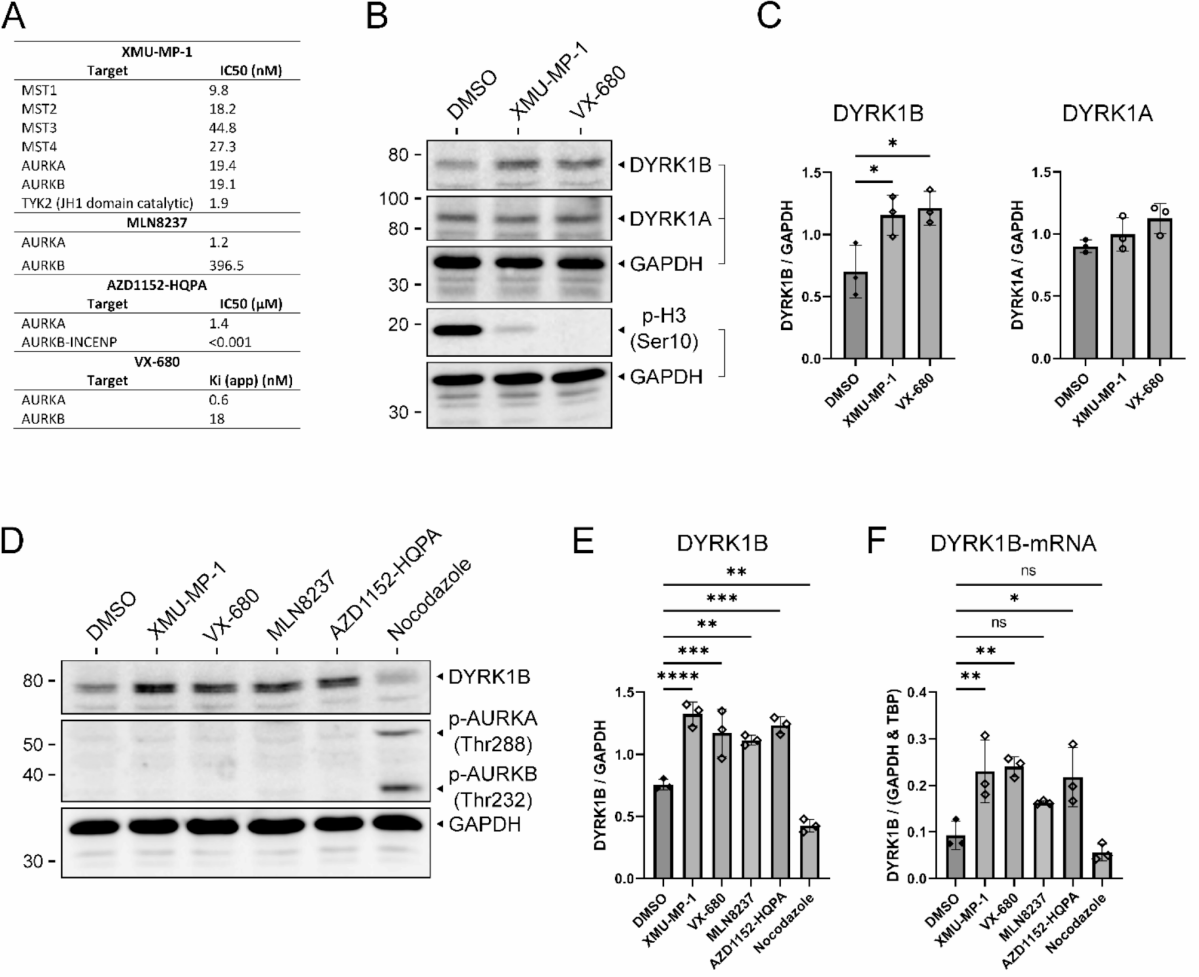
Treatment with Aurora kinase inhibitors upregulates DYRK1B expression in A549 cells. (**A**) The table lists IC_50_ or Ki values of kinase inhibitors for AURK and potential off targets of XMU-MP-1. Data were taken from the following sources: XMU-MP-1^27^, MLN8237^29^, AZD-HPQA^32^, VX-680^33^. (**B-C**) Representative Western blot and densitometric analysis of A549 cells treated with 3 µM XMU-MP-1, 2 µM VX-680 or DMSO 24 h prior to lysis. Phosphorylation of histone H3 was detected on parallel gels with 15% acrylamide. (**D-E**) Representative Western blot and densitometric analysis of A549 cells treated with 3 µM XMU-MP-1, 2 µM VX-680, 50 nM MLN8237, 300 nM AZD1152-HQPA, 100 ng/ml Nocodazole or DMSO as a negative control for 24 h prior to lysis. (**F**) qPCR analysis of experiments with conditions identical to (D-E). All data are shown as means ± SD (*n* = 3). Statistical analysis by One-way ANOVA and Dunnett’s multiple comparison test as a post-hoc-test (* *p* < 0.05; ** *p* < 0.01; *** *p* < 0.001, **** *p* < 0.0001 individual value if *p* < 0.1, ns *p* > 0.1).

## Treatment with Aurora kinase inhibitors upregulates DYRK1B expression in A549 cells

To further investigate the mechanism by which XMU-MP-1 increases DYRK1B levels in A549 cells, we considered off-target effects of XMU-MP-1 on protein kinases other than MST1/2. In the original study by Fan et al.^27^. , three kinases in addition to MST1-4 were found to be inhibited by XMU-MP-1 at similar concentrations: Aurora kinase A (AURKA), Aurora Kinase B (AURKB) and TYK2 (Tyrosine kinase 2) (Fig. 5A). To investigate whether DYRK1B expression is controlled by AURK activity, A549 cells were seeded at low density and treated for 24 h with either the pan-AURK inhibitor VX-680 (a.k.a. Tozasertib), XMU-MP-1, or vehicle (DMSO) (Fig. 5B-C). DYRK1B expression was upregulated following treatment with either XMU-MP-1 or VX-680, while DYRK1A levels remained unaffected. Phosphorylation of histone H3 on Ser10 was analysed to monitor cellular AURK activity^28^. Treatment with XMU-MP-1 nearly eradicated the pH3(Ser10) signal, indicating effective inhibition of AURK activity at this concentration.

We proceeded to investigate the impact of AURK on DYRK1B expression by using inhibitors that target either AURKA or AURKB. To ensure selectivity, it is crucial to employ optimal concentrations of the inhibitors. MLN8237 (a.k.a. Alisertib) was used for selective inhibition of AURKA^29^. The optimal concentration of MLN8237 was determined through a dose-response experiment inspired by de Groot et al.^30^. (supplementary Fig. S7A). AZD1152-HQPA (a.k.a. Barasertib-HQPA or Defosbarasertib, a cell-permeable active metabolite of Barasertib) displays > 1,000-fold selectivity for AURKB over AURKA and was used at a concentration of 300 nM, which does not diminish cellular AURKA activity^30^. Nocodazole was used to arrest cells in G2/M-phase, when AURK activity is maximal^31^.

As shown in Fig. 5D-E, treatment with either of the AURK inhibitors led to increased DYRK1B levels. Nocodazole induced the activation of both AURKA and AURKB, as evidenced by the detection of the activating phosphorylation on Thr288 or Thr232 and resulted in reduced DYRK1B levels compared to the DMSO control. Identical experiments were conducted for qPCR analysis and confirmed the effect of AURK inhibition on DYRK1B mRNA levels (Fig. 5F).

These results suggest that the effect of XMU-MP-1 on DYRK1B expression can likely be attributed to its inhibitory action on AURK.

## Discussion

DYRK1A and DYRK1B share similar functions in cell cycle regulation and have been implicated in the regulation of oncogenic processes^8,14,34,35^. However, the unbiased analysis of differentially expressed genes in human tumour samples has revealed that the expression of DYRK1B, but not DYRK1A, is elevated in various types of solid tumours^8^. Our present study has identified various conditions under which DYRK1B is upregulated in cultivated cancer cells. Increased expression of DYRK1B was observed upon serum-starvation, in cells grown to confluence, and upon treatment with AURK inhibitors on both protein and mRNA level. Importantly, DYRK1A remains mostly unaffected by these treatments, indicating that the expression of DYRK1A and DYRK1B is regulated by different mechanisms.

Serum deprivation is well known to upregulate DYRK1B expression in various cell lines^11,13,19,36^. Here we show that DYRK1B levels also increase when cells are grown to confluence in the continuous presence of serum. To the best of our knowledge, density-dependent regulation of DYRK1B expression has so far only been documented in murine NIH3T3 fibroblasts^4^. That study reported a reduction of DYRK1B levels in fibroblasts that were first maintained at confluence for 3 days and then re-plated at lower density with fresh medium. In this protocol, the increased DYRK1B levels in confluent cells may result from nutrients and growth factor depletion, the accumulation of metabolites or secreted factors, or changes in acidity. The present experiments were designed to minimize potential confounding factors, with cells being grown synchronously at different densities and media changed daily (Fig. 3A-E). While the influence of other factors cannot be entirely ruled out, we consider cell-cell-contacts to be the most likely stimulus for DYRK1B upregulation. It is conceivable that the observed upregulation of DYRK1B expression in 3D culture compared to 2D monolayer culture^36–38^ can be partially attributed to the extensive cell-cell contacts in tumour cell spheroids. However, it must be acknowledged that other factors in the complex microenvironment in the core of the spheroid may also play a role^39^.

We attempted to utilize the purported MST1/2 inhibitor XMU-MP-1^27^ to elucidate the contribution of the Hippo pathway to the density-dependent regulation of DYRK1B expression. However, treatment with XMU-MP-1 hardly affected the phosphorylation of YAP on Ser127 in the cell models used in our study (Fig. 4D, E). Consequently, no conclusions can be drawn regarding the regulation of DYRK1B levels by the Hippo pathway. We utilized the same concentrations of XMU-MP-1 that were previously used to reduce the phosphorylation of YAP in HepG2 cells^27^. Consistent with our negative result, XMU-MP-1 also proved ineffective in reducing the fraction of phosphorylated Yap in mouse utricules^40^ and failed to increase the active, unphosphorylated form of YAP in human hair follicle cells^41^.

We serendipitously discovered that XMU-MP-1 seemed to heighten DYRK1B expression through off-target inhibition of Aurora kinases. The effective inhibition of AURK is demonstrated by the effect on histone H3 phosphorylation on Ser10 (Fig. 5B), a known target site of AURK^42,43^. Reduced H3(Ser)-10 phosphorylation, indicative of off-target AURK inhibition by XMU-MP-1, has been observed in a human hair follicle organ culture model as well^41^. While the effects of XMU-MP-1 may vary depending on the context, the capacity of efficient AURK inhibition raises doubts about its proposed use as a MST1/2 inhibitor and a tool for investigating of the Hippo pathway.

We used three chemically divergent and well established inhibitors of AURK to corroborate the hypothesis that AURK function as upstream regulators of DYRK1B expression. AURKA and AURKB are serine/threonine kinases with pivotal roles in mitosis^44^. They share high sequence similarity^44^ and phosphorylation site motifs^45^. Despite these similarities, they exert different tasks during mitosis, which can be attributed to their divergent subcellular localization^46^. Aurora kinases are extensively studied as drug targets in lung cancer therapy^47^ and various other cancer entities^48^. Several AURK inhibitors including MLN8237 (Alisertib) and AZD1152-HQPA (Barasertib) have entered clinical trials^49,50^.

The potential link between DYRK1B expression and AURKA/B activity has not been previously reported. Notably, the upregulation of DYRK1B after treatment with MLN8237 and AZD1152-HQPA is not simply a consequence of cell cycle arrest, as evidenced by the effect of Nocodazole. Nocodazole is an anti-mitotic agent that arrests cells in M phase, but led to reduced levels of DYRK1B rather than an increase (Fig. 5).

The AURK inhibitors affected DYRK1B expression on the level of mRNA abundance. DYRK1B has been previously reported to undergo transcriptional upregulation following mTORC inhibition^19^. We confirmed this effect in PANC-1 and A549 cells with the help of the selective mTORC1/2 inhibitor KU-0063794. Interestingly, AURKA has been reported to activate the PI3K/AKT/mTOR-pathway^51^, and silencing or inhibition of AURKA/B suppresses mTORC activity in various cell lines^51–54^. It appears plausible that the observed effect of AURK inhibitors on DYRK1B expression cells may be indirectly mediated through mTORC inhibition. Regardless of the mechanism, the upregulation of DYRK1B in response to AURK or mTORC inhibitors may contribute to the development of cancer cell resistance to chemotherapeutic drug treatment^50^.

A systematic comparison between DYRK1A and DYRK1B expression has not yet been reported. Our data show that a variety of factors including serum deprivation, cell density or pharmacological intervention by KU-0063749, XMU-MP-1, MLN8237 and AZD1152-HQPA upregulated DYRK1B expression, while DYRK1A remained mostly unaltered or was even inversely affected. This differential response to stimuli that are potentially relevant in human tumours correlates with the observation that DYRK1B, but not DYRK1A, was found to be upregulated in many human cancer samples in the TCGA database^8^.

Consistent with previous studies^19,55^, we observed concordant changes of *DYRK1B* mRNA and protein in response various of stimuli. These findings suggests that the cellular abundance of DYRK1B is regulated by either the rate of transcription or mRNA degradation. Due to the methodological limitations of our reporter gene assays, which analysed putative promoter sequences by transient transfection outside the appropriate chromatin context, we can neither confirm nor refute the possibility that *DYRK1B* gene expression is controlled by promoter elements proximal to the transcription initiation site.

Several methodological limitations must be recognized in the interpretation of the present results. The effects of XMU-MP-1 and the AURK inhibitors were only observed in the A549 cell line, and it remains to be shown whether other cell lines also show this phenomenon. While AZD1152-HQPA and MLN2387 show relative selectivity towards AURKA and AURKB, the use of these inhibitors does not definitely prove the involvement of the individual isoforms. Our result do not mechanistically link effect of AURKs in DYRK1B expression with physiological conditions such as serum starvation, hypoxia, or high cell-density. Finally, it must be noted that the densitometric evaluation of Western blot experiments is a semi-quantitative method that does only provide an approximate measure of the changes in protein abundance.

## Methods

### Cell culture

PANC-1 cells were kindly provided by Edgar Dahl, Institute of Pathology, University Hospital RWTH Aachen University, Germany. OVCAR-3 cells were purchased from CLS Cell Lines Service, Eppenheim, Germany (#300307). A549 cells and SH-SY5Y cells were available in the department from previous studies^3,56^. PANC-1, A549 and SH-SY5Y cells were cultivated in DMEM/F12, Hepes (Gibco 11330-032). OVCAR-3 cells were cultivated in RPMI 1640 (Pan Biotech #P04-18047). All media were supplemented with 10% FBS (Gibco fetal bovine serum #10270106) if not stated otherwise. Identity of cell lines was verified by Multiplex human Cell line Authentication Test (MCA; Multiplexion GmbH) or confirmed by the provider (OVCAR-3). For seeding into multiwell plates, cells were washed with PBS once, trypsinized and counted in Neubauer chambers. The number of cells plated is given in supplementary Table S1. Pharmacological treatments were carried out 24–25 h after plating if not stated otherwise.

### Materials

Sources and identification of commercially purchased bioactive compounds and antibodies are listed in Tables S2 and S3 in the supplementary material.

### Immunoblot analysis

Cells were washed with PBS once and SDS buffer (1% SDS, 20 mM Tris-HCl; preheated at 100 °C) was applied to the wells. Lysates were collected by scraping, transferred to micro test tubes, and then incubated for 5 min at 100 °C. Afterwards lysates were vortexed, sonicated twice for 45 s with a 15 s interval and vortexed again. Insoluble cell debris was removed by centrifugation for 13 min at 14.000 rpm. Protein concentrations were determined using the Pierce BCA Protein Assay Kit (Thermo Scientific #23225) and BSA (PAA Laboratories GmbH #K51-001) as a standard. Lysates were diluted 1:5 with SDS buffer prior the assay to ensure they fell within the determination range of the assay. Samples were adjusted to suitable concentrations to allow analysis of equivalent amounts of protein by SDS polyacrylamide gel electrophoresis analysis and Western blotting. The Biotinylated Protein Ladder Detection Pack (CST #7727) was used as size standard. Membranes were blocked in TBST supplemented with 5% BSA overnight at 4 °C or at room temperature for at least 1 h. For immunodetection, membranes were incubated with the indicated antibody in blocking solution overnight at 4 °C or at room temperature for at least 1 h after washing them 3 times in TBST. Afterwards membranes were washed again 3 times in TBST and incubated in secondary antibody solution (5 µl HRP-coupled secondary antibody; 10 ml TBST supplemented with 5% BSA; 10 µl anti biotin HRP-coupled antibody) for at least 1 h at room temperature. Membranes were washed again 3 times in TBST, covered in detection solution and chemiluminescence was captured with a LAS-3000 CCD detection system (Fujifilm). Only exposures in the linear range of the detector were used for densitometric analysis.

Densitometric analysis was performed with the Multi Gauge Software (Fujifilm). Detected bands were marked manually and individual local background was determined within 10 pixels above and below the individual band. If interfering signals were present in this area, the background detection was only applied to the undisturbed area. The local background was then calculated relative on pixel size of the background areas and applied to the size of the detected band. Because biological replicates occurred on different western blots, densitometric analysis of each sample was normalized as a ratio to the sum of all signals of a biological replicate on a blot within one detection to ensure comparability between different blots^57^. GAPDH served as a loading control and the expression values of each protein were calculated by division of the signal of interest by GAPDH.

### qPCR

Cells were washed with 2 ml PBS once, scraped in 1 ml room-temperature PBS and centrifuged with 300 x g for 5 min. Cell pellets were stored at -70 °C until RNA was isolated from frozen pellets using the QIAcube robotic workstation and the RNeasy Mini Kit (Qiagen #74104), QIAshredder (Qiagen #79654) and the RNase-Free DNase Set (Qiagen #79254) for on-column DNase Digest. RLT Buffer supplemented with 20 mM DTT was applied to the frozen pellets, and the lysates were homogenized by passaging through a 20-gauge needle 6 times. RNA concentrations were quantified photometrically (NanoDrop ND-1000, Peqlab). RNA was reverse transcribed using the PrimeScript RT Reagent Kit (Takara #RR037A) following the manufacturer’s instructions. Equal amounts of RNA from each sample of a biological replicate were used for reverse transcription, with a maximal RNA concentration of 50 ng/µl. No-template controls (NTC) and no-reverse-transcriptase controls (NRT) were run in all experiments. qPCR was performed using the LightCycler 480 SYBR Green I Master System (Roche #04707516001) following the manufacturer’s instructions on a CFX Connect Real-Time PCR Detection System (Biorad). Primer design, choice of reference genes and thermocycling parameters are detailed in the supplementary material (Tables S4 and S5). Criteria of quality control and details of data analysis are given in the supplementary material. An alternative qPCR protocol was used to re-analyse RNA samples from experiments that did not meet the quality criteria (Figs. 4H-I and 5F). This protocol is provided in the supplementary material. TBP and GAPDH were identified as suitable reference genes for relative quantitation of mRNA levels by using CFX Maestro Software 1.1 (Bio-Rad).

### Statistics and image processing

Statistics were carried out using Graph Pad Prism 10.1.2–10.2.0 for Windows (GraphPad Software, Boston, Massachusetts USA). Data were tested for normal distribution by the Shapiro Wilk test as indicated in the Figure legends. Graphics were created using the Inkscape Software (v1.1, www.inkscape.org), GraphPad Prism 9 and 10.2.0, and GIMP (v2.10.22, www.gimp.org).

## Electronic supplementary material

Below is the link to the electronic supplementary material.

Supplementary Material 1

## Data Availability

All relevant data of this study are available within the article and its supplementary information.

## Abbreviations

AURK: Aurora kinases
DMSO: Dimethyl sulfoxide
FBS: Fetal bovine serum
HRP: Horseradish peroxidase
mTORC1: Mechanistic target of rapamycin complex 1
NSCLC: Non-small-cell lung cancer
qPCR: Quantitative real-time polymerase chain reaction
PBS: Phosphate buffered saline
