# Supplementary material for: Differential regulation of expression of the protein kinases DYRK1A and DYRK1B in cancer cells
Source: Sci Rep. 2024 Oct 13;14:23926. doi: 10.1038/s41598-024-74190-1 (PMC11471791; doi:10.1038/s41598-024-74190-1)

**Supplementary Information**

# Differential Regulation of Expression of the Protein Kinases DYRK1A and DYRK1B in Cancer Cells

Vincent Andreas Vorwerk[1], Gerrit Wilms[1], Aaron Babendreyer[2], Walter Becker[1]

[1]*Institute of Pharmacology and Toxicology, RWTH Aachen University, 52074 Aachen, Germany*

[2]*Institute of Molecular Pharmacology, RWTH Aachen University, 52074 Aachen, Germany*

## Content:

1. **Supplementary materials and methods**
2. **Construction of the reporter gene plasmids**
   Fig. S1: Human DYRK1B promoter sequences inserted into pGL3 basic
3. **Supplementary results**
   Fig. S2: Separate densitometric quantification of Fig. 2
   Fig. S3: Differential expression of DYRK1B splicing variants in cancer cell lines
   Fig. S4: Differential promoter usage of the DYRK1B gene in cancer cell lines
   Fig. S5: Analysis of the human DYRK1B promoters under serum depletion
   Fig. S6: Pilot experiment for optimization of XMU-MP-1 treatment
   Fig. S7: Dose finding experiments for selective inhibition of AURKA or AURKB
4. **Supplementary References**
5. **Uncropped Western blots**

# 1. Supplementary materials and methods

## Cell culture

**Table S1**: Conditions of cell plating

| Figure | | Cell line | Cells plated | Plates used |
|---|---|---|---|---|
| 1 | | | 150.000 | 6 Well Plate |
| 2 | | | 125.000 | 6 Well Plate |
| 3 | | | As indicated | 6 Well Plate |
| 4 | | | 150.000 | 6 Well Plate |
| 5 | | | 150.000 | 6 Well Plate |
| S3B | | | not determined | 6 Well Plate |
| S4 | B | PANC-1 | 125.000 | 12 Well Plate |
| | | A549 | 62.500 | |
| | | OVCAR-3 / SH -SY5Y | 250.000 | 6 Well Plate |
| S5 | | | 90.000 | 6 Well Plate |
| S6 | A | | As indicated | 6 Well Plate |
| | B-C | | 150.000 | 6 Well Plate |
| S7 | A | | 250.000 | 6 Well Plate |
| | B | | 150.000 | 6 Well Plate |

## Materials

**Table S2** Bioactive compounds

| Compound | Source | Order Number |
|---|---|---|
| XMU-MP-1 | Sigma-Aldrich | #SML2233 |
| VX-680 (Tozasertib) | LC Laboratories | #T-2304 |
| MLN8237 (Alisertib) | MedChemExpress | #HY-10971 |
| AZD1152-HQPA (Barasertib-HQPA) | MedChemExpress | #HY-10126 |
| KU-0063749 | Selleckchem | #S1226 |

**Table S3** Antibodies

| Target | Primary Antibody | RRID[a] |
|---|---|---|
| DYRK1B | Cell Signaling Technology #2703 | RRID:AB_2261790 |
| DYRK1A | Sigma-Aldrich #D1694 | RRID:AB_1840821 |
| GAPDH | Cell Signaling Technology #2118 | RRID:AB_561053 |
| YAP | Santa Cruz #sc-101199 | RRID:AB_1131430 |
| p-YAP (Ser127) | Cell Signaling Technology #4911 | RRID:AB_2218913 |
| p-4-EBP-1 | Cell Signaling Technology #2855 | RRID:AB_560835 |
| p-Aurora A (Thr288) / Aurora B (Thr232) / Aurora C (Thr198) | Cell Signaling Technology #2914 | RRID:AB_2061631 |

| Target | Secondary Antibody | |
|---|---|---|
| Rabbit IgG | Rockland #611-703-127 | RRID:AB_218614 |
| Mouse IgG | Rockland #610-703-124 | RRID:AB_218548 |
| Biotin | Cell Signaling Technology #7075 | RRID:AB_10696897 |

[a] Research Resource Identification Portal (https://scicrunch.org/resources)

## Oligonucleotides and conditions of qPCR

Primer for DYRK1B were designed using the PerlPrimer Software [1] (v1.1.21) to detect all 3 known splice-variants DYRK1B-p69/-p75/-p65 described by Leder et al.[2]. Primer for DYRK1A have already been designed and verified in a previous study [3]. Primer sequences are described in Table S4 and were obtained from Eurogentec. Primer sequences for GAPDH and TBP had been established previously [4]. The optimal annealing temperature for DYRK1A and DYRK1B primers were established by gradient PCR ranging from 55°C to 65°C. For DYRK1B primers, a dilution series qPCR using the optimal temperature was performed, and specificity of the qPCR product and absence of primer-dimers were verified by agarose gel electrophoresis. All melting curves of all qPCRs performed under these optimised conditions were checked for primer-dimers and found negative.

qPCR reactions were run in total volume of 10 µl. Plastic materials for qPCR were obtained from BioPlastics. cDNA samples were diluted 1:6 prior to qPCR. Thermocycling parameters can be seen in table S5.

**Table S4:** Oligonucleotide primers used for qPCR

| Target | Forward Primer | Reverse Primer | Annealing Temperature |
|---|---|---|---|
| **hDYRK1B** | GATCTACCAGTATATCCAGAGCC | CCCTGGTAATCCTTCCTGAG | 59 °C |
| **hDYRK1A** | TCTGGGTATTCCACCTGCTC | GTCCTCCTGTTTCCACTCCA | 63.3 °C |

° Homo sapiens specific

**Table S5:** Thermocycling conditions

| | | |
|---|---|---|
| **Initial Denaturation** | | |
| 1x | 95 °C | 5 min. |
| **Cycling** | | |
| 40 x | 95 °C | 10 sec. |
| | Individual annealing temperature | 10 sec. |
| | 72 °C | 15 sec. |
| | | Plate Read |
| **Melting Curve** | | |
| 65 °C to 95 °C with 0.5 °C increment and subsequent Plate Read | | |

### qPCR data analysis and criteria for quality control

Efficiencies of qPCR-runs were calculated using the LinRegPCR software (Version 2020.2) [5]. Data were analysed using the Bio-Rad CFX Maestro Software (Bio-Rad Laboratories, Version 4.1.2433.1219). Cq values were determined using the single threshold determination method. For reference gene selection, qPCR was performed using a series of 8 reference genes and samples from PANC-1 cells (Fig. 1C and 3F). Data were analysed using the GeNorm algorithm [6] and GAPDH and TBP were chosen as the most stable reference genes. All results were normalized to both reference genes using the ΔΔCq-algorithm of the Bio-Rad CFX Maestro Software. qPCR reactions were run as triplicates (Fig. 1K) or duplicates (Fig. 1E,F,L and Fig.

3F,G). In experiments with 3 replicates, single data points were excluded when exceeding an intra-assay variation of 1 Cq. No values were excluded when PCRs were run in duplicate.

qPCRs results were considered as technically not reliable and therefore excluded from analysis, if (a) exceeding the mean intra-assay variation of 1 Cq or (b) too high Cq-Values within the range of the negative controls or (c) intra-assay variation greater than 1 Cq between all 3 technical replicates. Consequently, cDNA synthesis and qPCR analysis of these RNA samples were repeated by using the "alternative qPCR protocol" (see below).

### Alternative qPCR protocol

The alternative qPCR protocol was used to re-analyse RNA samples from the experiments from Figure 4H-I and 5F, which did not meet the quality criteria as described above. Reverse transcription was carried out using the Maxima Reverse Transcriptase, Oligo(dt)18 Primer and RiboLock RNase Inhibitor from Thermo Scientific as well as an dNTP mix manually mixed out of stock solutions obtained from Peqlab following the manufacturer's instructions with the modified incubation of 10 minutes at 25 °C followed by 15 minutes at 50 °C and termination at 85 °C for 5 minutes. cDNA samples were diluted 1:1 prior to qPCR. qPCR was performed using the LightCycler 480 SYBR Green I Master System (Roche #04707516001) on a LightCycler 480 System (Roche). Data were analysed using the LightCycler® Software (Roche) and efficiency was calculated by analysis of a cDNA dilution series. Results were normalised to GAPDH and TBP separately. Values were then calculated as a ratio to the total of all samples of a biological replicate combined. Data shown were then defined as the mean of the target gene normalized by GAPDH and the same target gene normalized by TBP.

### Reporter gene assay

Cells were co-transfected the next day after plating in 6–well plates using FuGENE HD (Promega) (Fugene : DNA ratio 3:1) with 400 ng of the pGL3 reporter plasmid and 100 ng of the phRG-TK plasmid (Promega #E6291) as a control for transfection efficiency (266 ng reporter plasmid DNA and 66 ng phRG-TK Renilla plasmid in 12-well plates). Media were changed one day after transfection. Cells were washed once with PBS and lysed using 1x Passive Lysis Buffer (Promega #E194A). Plates were agitated on a shaker for 15 min before the lysates were collected by scraping and transferred into micro test tubes. Lysates were vortexed and cell debris was removed by centrifugation (2 min, 14,000 x g). Aliquots of 10 µl of the supernatants were transferred to 96–well plates and equilibrated to room temperature prior to performing the luciferase assays with a 7.5 s delay before 5 s luminescence measurement (Berthold Detection Systems Microplate Luminometer MPL4). The Firefly Luciferase Assay System (Promega #E1501) was used following the manufactures instructions with 50 µl LAR I reagent. Assays of Renilla luciferase were performed in a buffer described [7] in a volume of 100 µl with Coelenterazine as substrate (PJK Biotech, Kleinblittersdorf, Germany (#102171)). Relative Luciferase Units (RLU) were calculated by division of firefly luminescence by Renilla luminescence.

## 2. Construction of reporter gene plasmids

### In silico promoter analysis

To define the position of the *FBL*/*DYRK1B* border, Expressed Sequence Tags were mapped to the area upstream of the human DYRK1B-exon 1A. For the purpose of this study, the end of the 3' noncoding *FBL* exon was arbitrarily considered as the border between the *FBL* gene and the promoter of the *DYRK1B* gene. Exon-intron borders of *DYRK1B* were defined based on cDNA sequences listed in Table S6. Putative promoter regions were determined by compiling transcriptions start sites using the CAGE-TAG data from the FANTOM5 Database [8,9]. Translation starts and splicing structure were taken from [2].

**Table S6** cDNA sequences used for the definition of the exon-intron structure in Fig. 2A

| | Fibrillarin Exon | DYRK1B Exon 1A | | Exon 1B | | Exon 2 | |
|---|---|---|---|---|---|---|---|
| Border (Antisense) | 3' | 5' | 3' | 5' | 3' | 5' | 3' |
| Reference | BU683240.1 | Bi561053.1 | | ENST00000600611.5 & ENST00000626964.2 | HY001857.1 | Bi561053.1 | |

### Construction of reporter gene plasmids

Promoter regions were inserted into pGL3-basic vector (Promega #E1751, GenBank accession #U47295) using the NEBuilder HiFi DNA Assembly System. Cloning was planned with the NEBuilder Assembly Tool with reconstruction of restriction sites. The pGl3-basic backbone was linearized with XhoI. Promoter regions were amplified from HEK293 genomic DNA using the primers listed in Table S7. For bacterial plasmid preparation the GeneJET Plasmid Mini-prep-Kit (Thermo Scientific #K050X) and Qiagen Plasmid Kit Maxi (Qiagen #12163) were used. Correct insertion was confirmed by plasmid sequencing (Eurofins Genomics) (Figure S1).

**Table S7**

| Construct | Forward Primer | Backward Primer | Insert Size |
|---|---|---|---|
| Promoter I | ttacgcgtgctagcccgggctcgag TCCCATGTCTGCTGCTCATTC | gcttacttagatcgcagatctcgag CTGAAGGAGCGGGCAAGG | 435 bp |
| Promoter II | ttacgcgtgctagcccgggctcga GTAACGGCGCGGGGACGG | gcttacttagatcgcagatctcga GCGGCGCTGAAAAGGCGAC | 667 bp |

## A)

## Promotor I

1 - TCCCATGTCTGCTGCTCATTCCTCCCCTTGACCTGCTGACACAGGGAGCACGCACCCTTG

61 - GTCAATTTTGCGGGGTTGGGTAAATTCTCACTCGGTCACAGAGCGCATGCTCCGTTTCTA

121 - GCTGCCTTTGCGCAGCGGCAGCCTGGATTTCGGTTCTTGGGTGGGATTGGTAGCTCGCTG

181 - CGCATGCGTGCAGGTAAGCGGCCATCTCGCGCAGGCGGAGTGTCAGTGTGGGTCACGTGA

241 - GGGGAGCGGAGAGGGAGGGATGGGGGCGGAGTCCAGGGCGTGGGGGGGCCGGTTTGTTGT

301 - GGTCGCCATTTTGCTGGTTGCATTACTGGGTAATCGGGGCCCTGGCTTGCCGCGTCCGCC

361 - GGATACCCTCAGCCAGTGGGCAGGTCTGAGCTCGGGCTCCCCGAGCAGTTTGAGTCCCCT

421 - TGCCCGCTCCTTCAG

100 % Identity & 0 Gaps aligned to GRCh38.p13 Primary Assembly, Chromosome 19

## B)

## Promotor II

1 - GTAACGGCGCGGGGACGGGTGGGGCGGCAAGCGGTCGCAGGGAGGTGGGCAGGACGGGAT

61 - CCGCCCTGCTCCCGTCGCCGTGAGACTTAGCACGAGGCCAAGGGAGGAGAGGAGGGGGGT

121 - GGCAGGCAGGTGCGGGCCCTGCCTGGCTATTCATAGTTGAATTCCTGGAACCGGCCAAGC

181 - CCGAGGAAGCAGTTGCAGGAGGGAGGCTGGGAGGGGGTAGCCGGGCCCCACTCCCGCCCT

241 - TTGTTTGGGCTCAGCTCCGCGGGCCGCTTCTTCGTCGCCTAGCAACAGCTGCCCTAGGCT

301 - GTGATTGGCTGAGCTCTTGGCACCAGCGACCAATGGTACAGTTGTTGCCATGGCAGGTGC

361 - CGATTGCCAAGCTCAGTCGGGCCCCGCCTTCCGGTCTCAGCAGGCCCAGGAGGGCCTCCT

421 - GGGT**A**GGGGGCGGGACGCCGGGTCCCTAGGGGCTGGTGGTCACTCAGGGTGGGGCGTGTC

481 - GCCCCTCCCCCGTCCACCTGCTCTACTCTTCCCCCGCGTGCCCTGGGCTGACCCTTGTCC

541 - CCTCCTCTCCCCGCCCCCGGTGGCAGTGGCGGCTGCTGTTGTCACCCACCGGGCCTCCTG

601 - TCCCGCTTGCCCTCCCCGCCGCGGGGCCGGCCGGGCCAGAGACAGGCGGTCGCCTTTTCA

661 - GCGCCGC

99 % Identity & 0 Gaps aligned to GRCh38.p13 Primary Assembly, Chromosome 19

**Figure S1. Human DYRK1B promoter sequences in inserted into pGL3 basic**
Sequences promotor constructs used in this study for promotor region I **(A)** and promotor region II **(B)**. One base in promotor region II deviates from the GRCh38.p13 genome assembly and is marked in red.

## 3. Supplementary Results

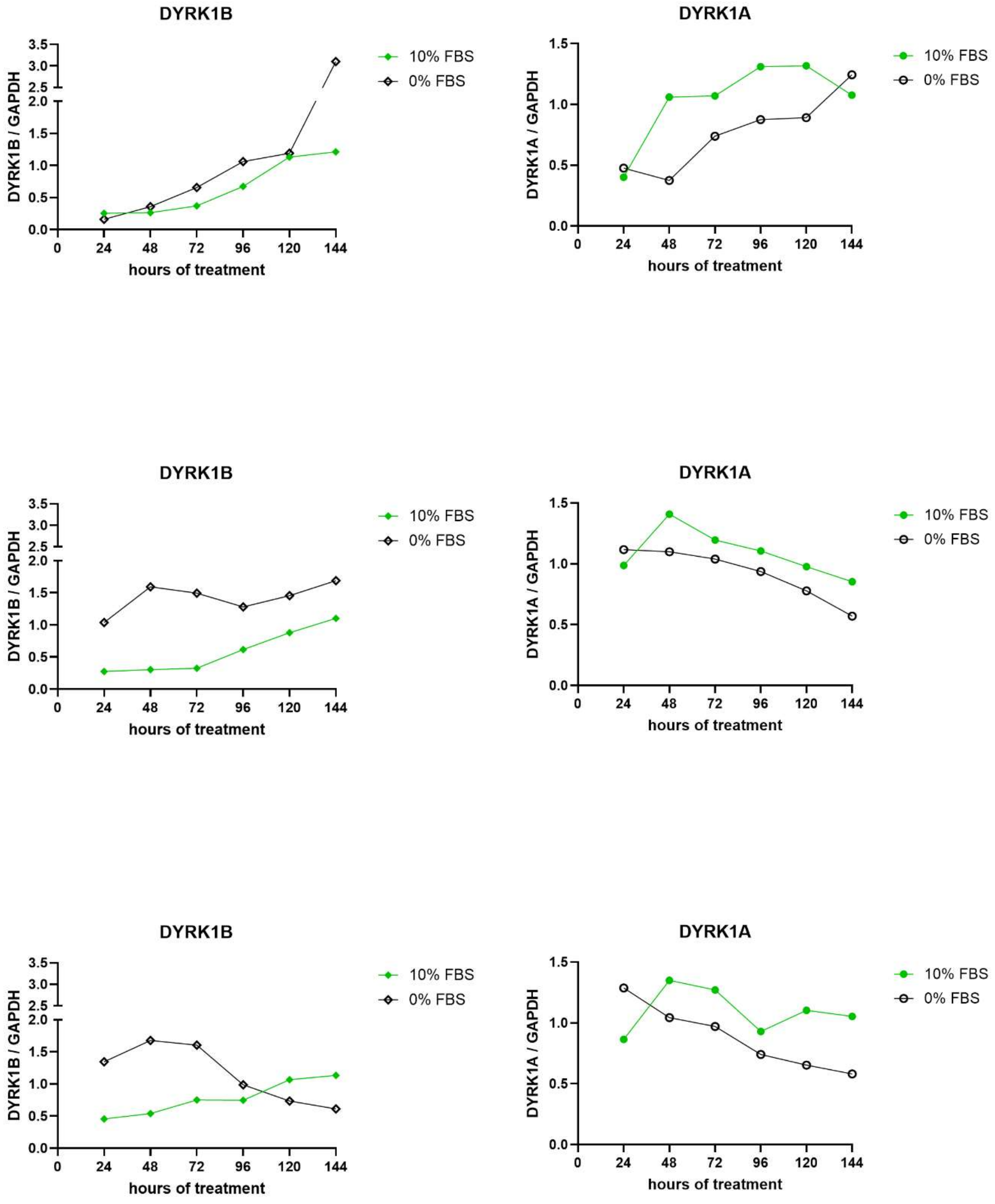


**Figure S2A. Separate quantification of the experiments in Figure 2A-B**

Each row represents a different biological replicate. Data is shown as individual data points of densitometric quantification of Western blots of Figure 2A.

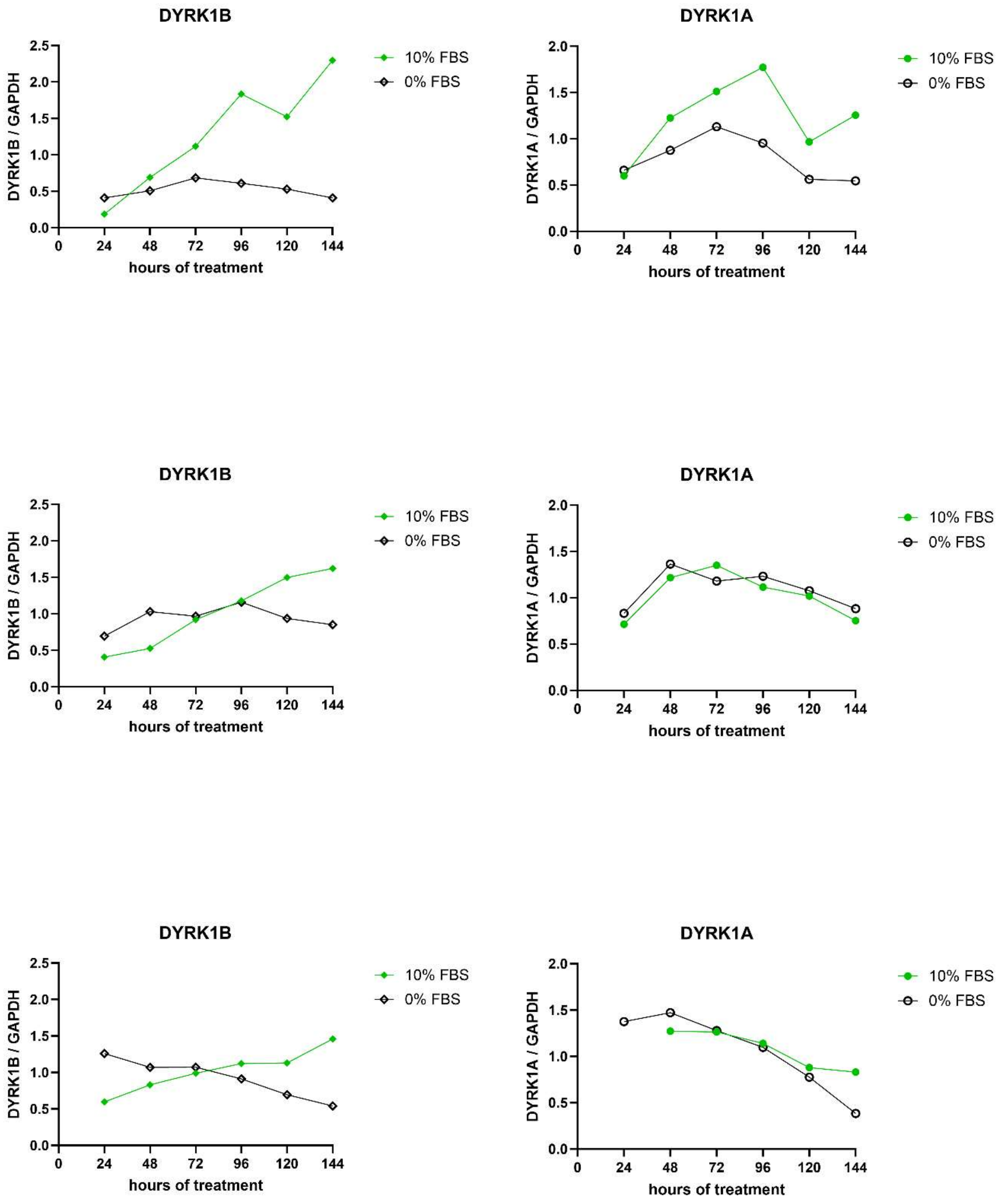


**Figure S2B. Separate quantification of the experiments in Figure 2C-D**

Each row represents a different biological replicate. Data is shown as individual data points of densitometric quantification of Western blots of Figure 2C.

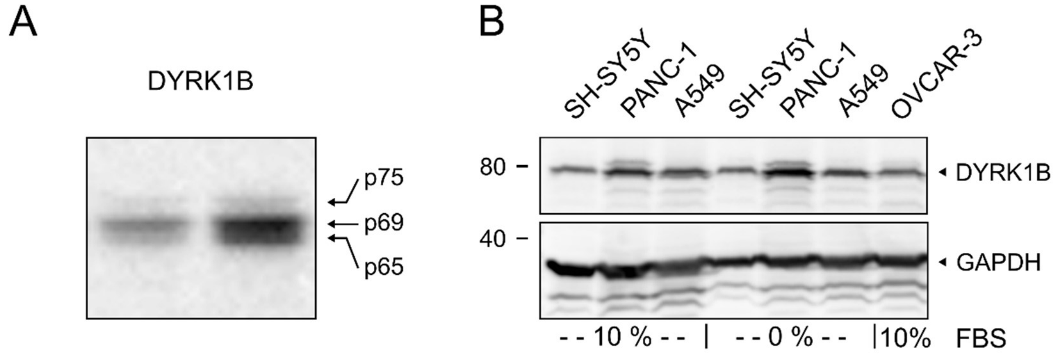


**Figure S3. Differential expression of DYRK1B splicing variants in cancer cell lines**
A) The image shows an enlarged cutout of Fig. 2A (72 h) to illustrate the presence of three splicing variants in PANC-1 cells. We have previously shown that p65 is a catalytically inactive form of DYRK1B that arises from alternative splicing between exons encoding the catalytic domain [2].p69 and p75 are generated by alternative usage of Exon 1A or Exon 1B (see Fig. S4).

B) Western blot analysis of DYRK1B in different cancer cell lines. Cells were grown to confluence and maintained for several days in the absence or presence of 10% fetal bovine serum before lysis.

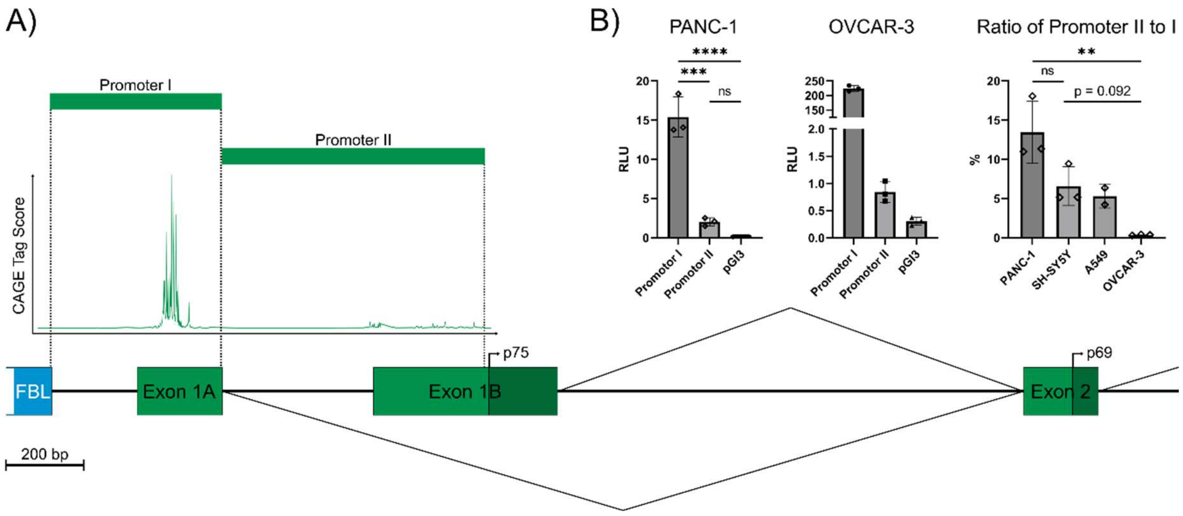


**Figure S4. Differential promoter usage of the *DYRK1B* gene in cancer cell lines.**

A) Schematic illustration of the *DYRK1B* promoter region. CAGE (cap analysis of gene expression) tag scores were taken from the FANTOM5 database and plotted onto the 5' region of the human *DYRK1B* gene. CAGE is a tool to map transcription start sites at single-nucleotide resolution [10]. Most transcription start sites map to the region between the 3'-end of the *FBL* (fibrillarin) gene and the 3'-end of *DYRK1B* exon 1A. This region was defined as promoter region I. The region upstream of the translational start codon of the p75 splicing variant showed a lower number of transcriptional starts and was termed promoter region II. Translation start sites of the splicing variants p69 and p75 are marked by arrows. The indicated promoter regions I and II were cloned into the vector pGL3-basic for luciferase-based reporter gene assays (Fig. S1).

**B)** Reporter gene activities of promotor I and II in different cancer cell lines. PANC-1, SH-SY5Y, OVCAR-3 and A549 cells were co-transfected with the indicated reporter constructs and a *Renilla* luciferase control plasmid 2 days before cells were lysed. Promoter activities were normalized to *Renilla* luciferase activity and are shown as Relative Luciferase Units (RLU). Ratios of promoter activities are shown as percent of promoter II activity relative to promoter I. All Data are shown as means ± SD; n = 3 except n=2 for A549. Statistical analysis by One-Way ANOVA with Bonferroni multiple testing correction as a post-hoc-test. Data of OVCAR-3 did not match the criteria of normality and were therefore not analysed for statistical significance (* $p < 0.05$; ** $p < 0.01$; *** $p < 0.001$, **** $p < 0.0001$, individual value if $p < 0.1$, ns $p > 0.1$). A549 cells were excluded from statistical analysis because of low sample size.

A)

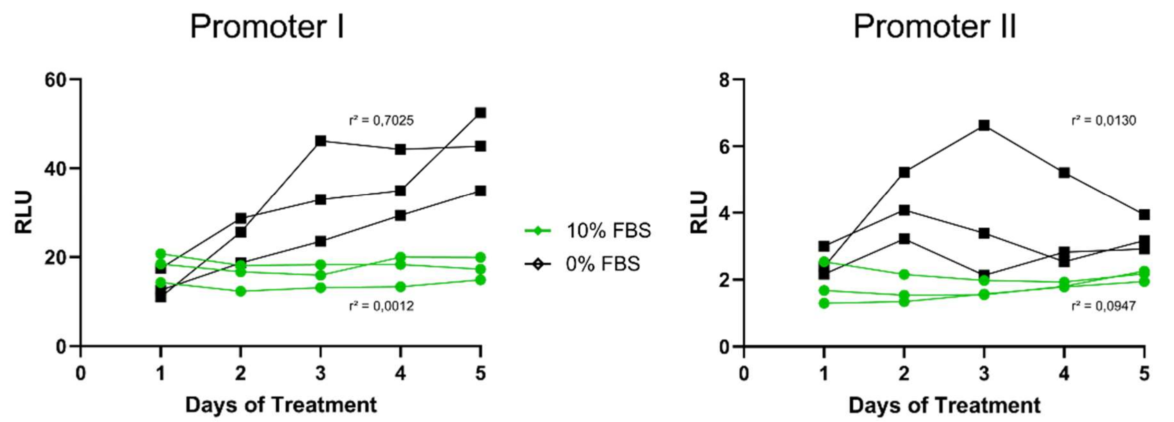


**Figure S5. Analysis of the human DYRK1B promoters under serum depletion**
PANC-1 cells were co-transfected with the indicated reporter construct and *Renilla* control plasmid one day after plating. Cells were treated with medium supplemented with either 10% FBS or 0% FBS on the next day. Lysis and change of cell media occurred daily for 5 days after treatment. The time course of promotor activity does not correlate with the changes on protein level, indicating that the cloned promoter fragments are not sufficient to mimic the transcriptional regulation of the *DYRK1B* gene under these conditions. Data are shown as individual data points (n = 3). Statistical analysis by linear regression model (multiple $r^2$ as indicated in the figure).

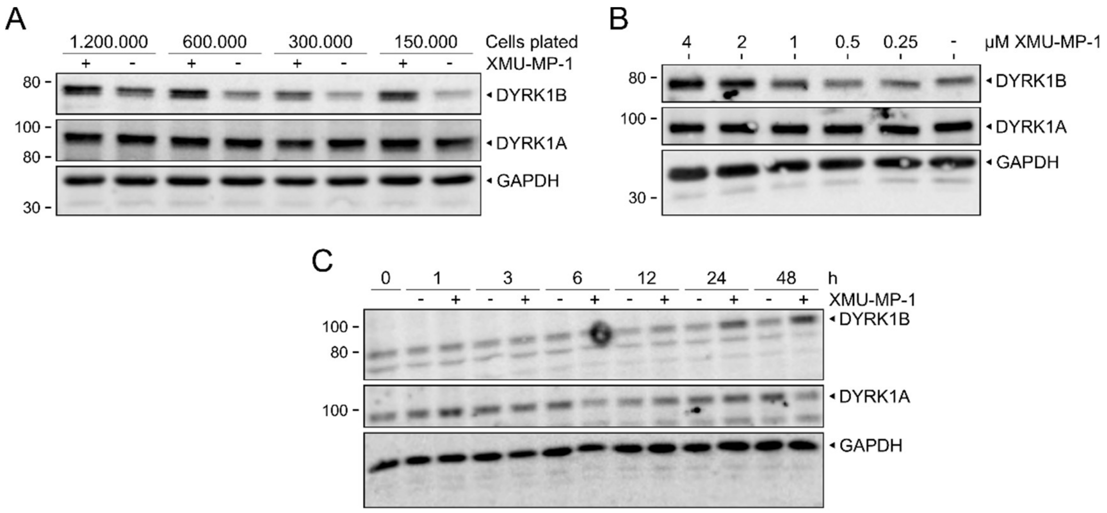


**Figure S6. Pilot experiment for optimization of XMU-MP-1 treatment**
**(A)** Western blot of A549 cells treated with either 3 µM XMU-MP-1 (+) or DMSO (-) for 24 h after plating at different cell densities and 24 h prior to lysis. **(B)** A549 cells were treated with different concentrations of XMU-MP-1 or vehicle only (-) for 24 h. **(C)** A549 cells were treated with 3 µM XMU-MP-1 (+) or DMSO (-) for variable times or left untreated (0 h).

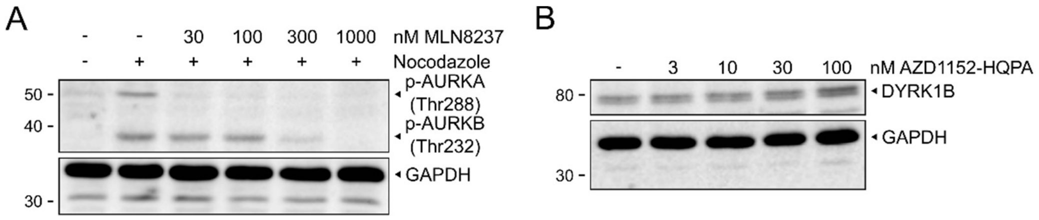


**Figure S7. Dose finding experiments for selective inhibition of AURKA or AURKB**
**(A)** A549 cells were treated with 100 ng/ml Nocodazole (+) and variable concentrations of MLN8237 for 16 h prior to lysis. (-) indicates treatment with the identical amount of DMSO for both Nocodazole and MLN8237 treatment. AURK inhibition was assessed by detection of autophosphorylation. A concentration of 50 nM was chosen to specifically inhibit AURKA without affecting AURKB. **(B)** A549 cells treated with the indicated concentrations of AZD1152-HQPA or DMSO (-) for 24 h

## 4. Supplementary References


1 Marshall, O. J. PerlPrimer: cross-platform, graphical primer design for standard, bisulphite and real-time PCR. *Bioinformatics* **20**, 2471-2472, doi:10.1093/bioinformatics/bth254 (2004).

2 Leder, S. *et al.* Alternative splicing variants of dual specificity tyrosine phosphorylated and regulated kinase 1B exhibit distinct patterns of expression and functional properties. *Biochem J* **372**, 881-888, doi:10.1042/BJ20030182 (2003).

3 Soppa, U. *et al.* The Down syndrome-related protein kinase DYRK1A phosphorylates p27(Kip1) and Cyclin D1 and induces cell cycle exit and neuronal differentiation. *Cell Cycle* **13**, 2084-2100, doi:10.4161/cc.29104 (2014).

4 Babendreyer, A. *et al.* The metalloproteinase ADAM15 is upregulated by shear stress and promotes survival of endothelial cells. *J Mol Cell Cardiol* **134**, 51-61, doi:10.1016/j.yjmcc.2019.06.017 (2019).

5 Ruijter, J. M. *et al.* Amplification efficiency: linking baseline and bias in the analysis of quantitative PCR data. *Nucleic Acids Res* **37**, e45, doi:10.1093/nar/gkp045 (2009).

6 Vandesompele, J. *et al.* Accurate normalization of real-time quantitative RT-PCR data by geometric averaging of multiple internal control genes. *Genome Biol* **3**, RESEARCH0034, doi:10.1186/gb-2002-3-7-research0034 (2002).

7 Inouye, S. *et al.* C6-Deoxy coelenterazine analogues as an efficient substrate for glow luminescence reaction of nanoKAZ: the mutated catalytic 19 kDa component of Oplophorus luciferase. *Biochem Biophys Res Commun* **437**, 23-28, doi:10.1016/j.bbrc.2013.06.026 (2013).

8 Lizio, M. *et al.* Gateways to the FANTOM5 promoter level mammalian expression atlas. *Genome Biol* **16**, 22, doi:10.1186/s13059-014-0560-6 (2015).

9 Lizio, M. *et al.* Update of the FANTOM web resource: expansion to provide additional transcriptome atlases. *Nucleic Acids Res* **47**, D752-D758, doi:10.1093/nar/gky1099 (2019).

10 Shiraki, T. *et al.* Cap analysis gene expression for high-throughput analysis of transcriptional starting point and identification of promoter usage. *Proc Natl Acad Sci U S A* **100**, 15776-15781, doi:10.1073/pnas.2136655100 (2003).

## 5. Uncropped Western Blots

### Figure 1A

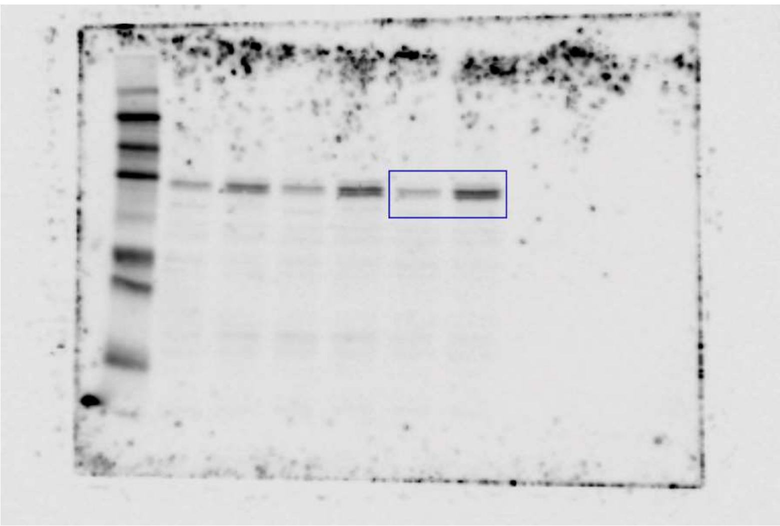

DYRK1A

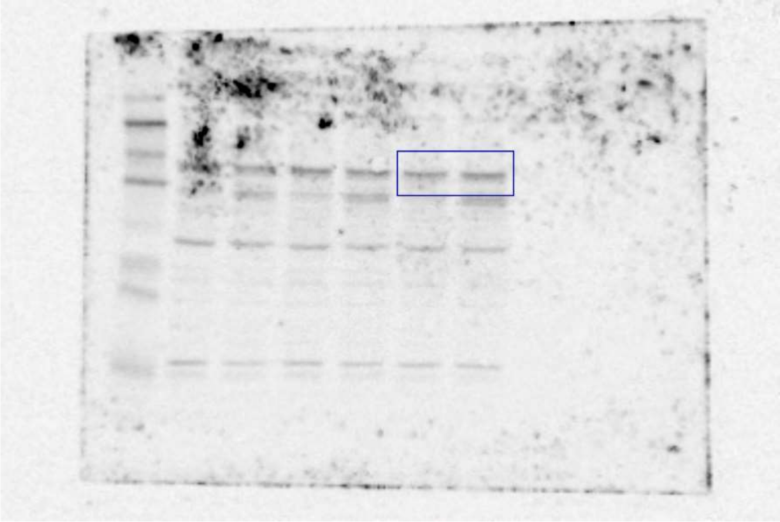

GAPDH

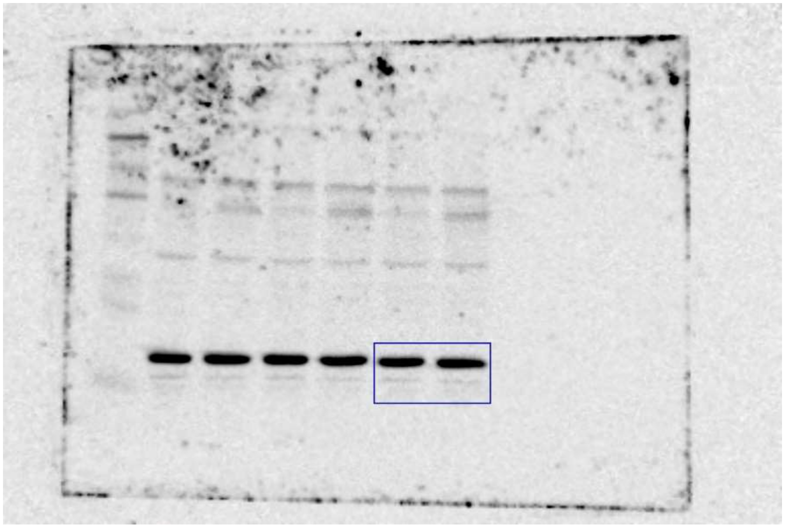

**Figure 1C**

DYRK1B

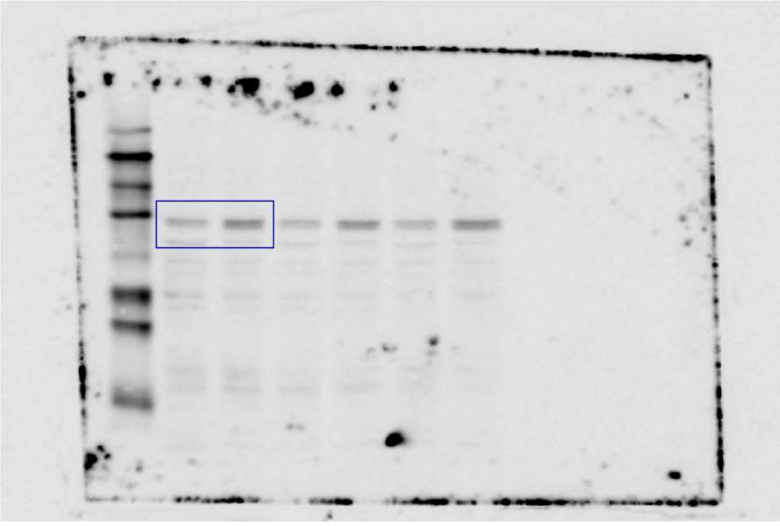

DYRK1A

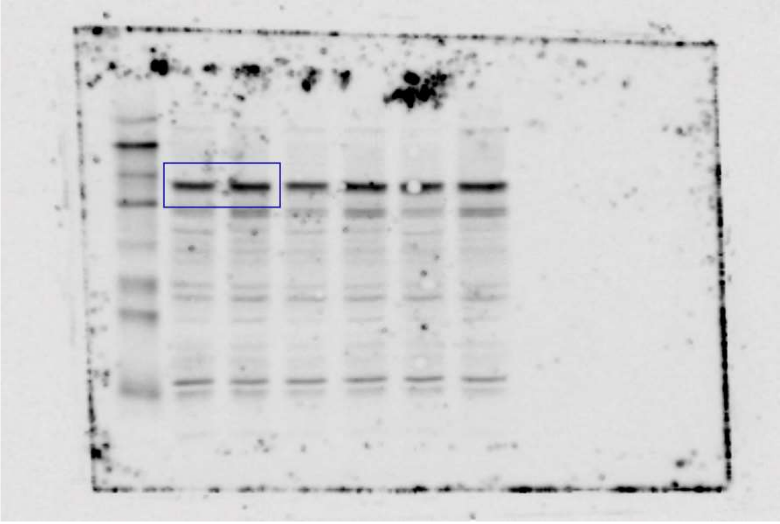

GAPDH

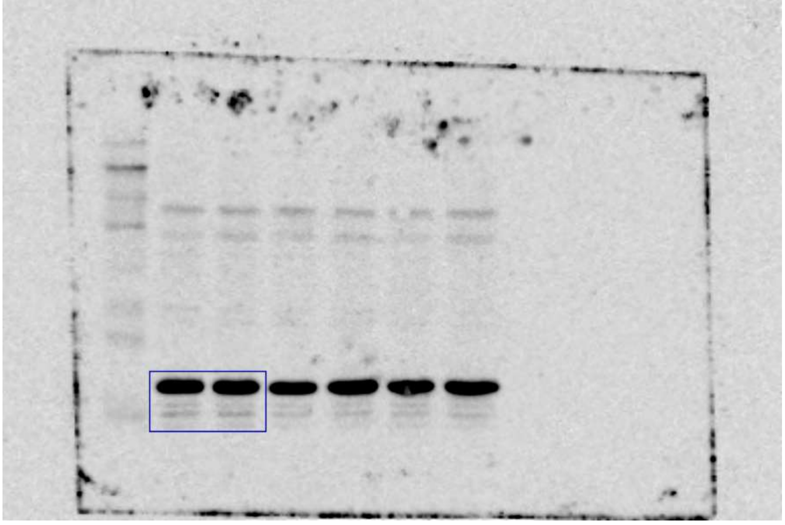

**Figure 1G**

DYRK1B

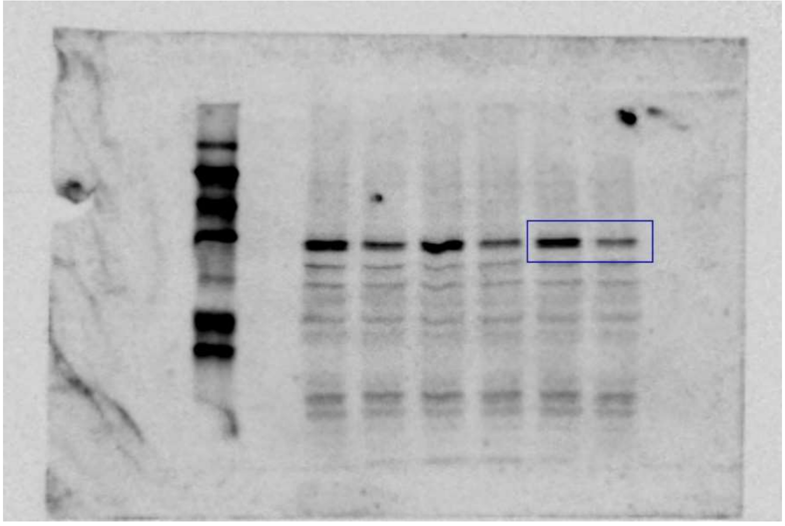

DYRK1A

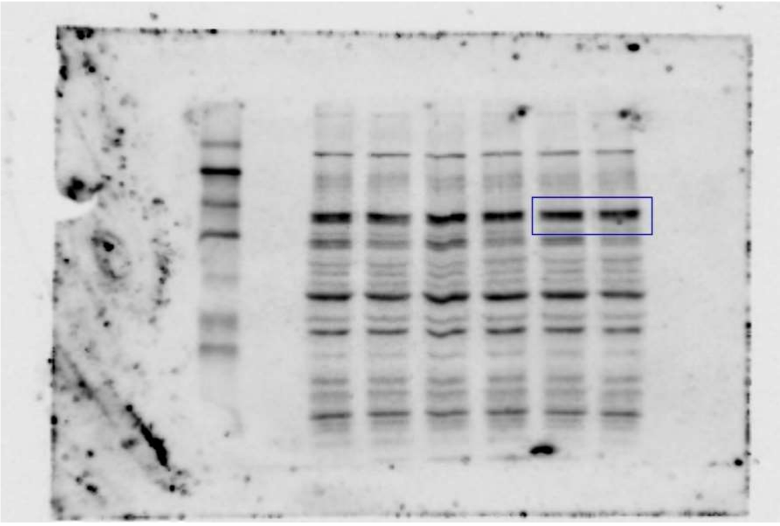

GAPDH

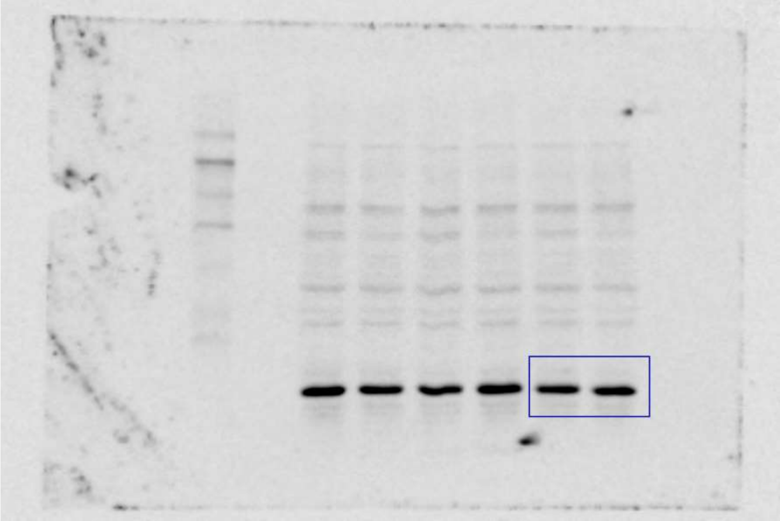

p-4E-BP1 (Thr37/46)

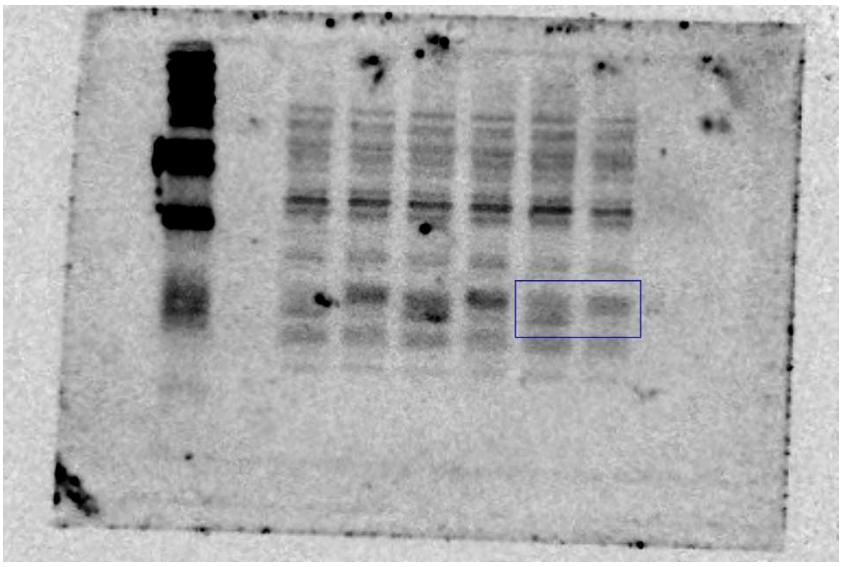

GAPDH (p-4E-BP1 – Blot)

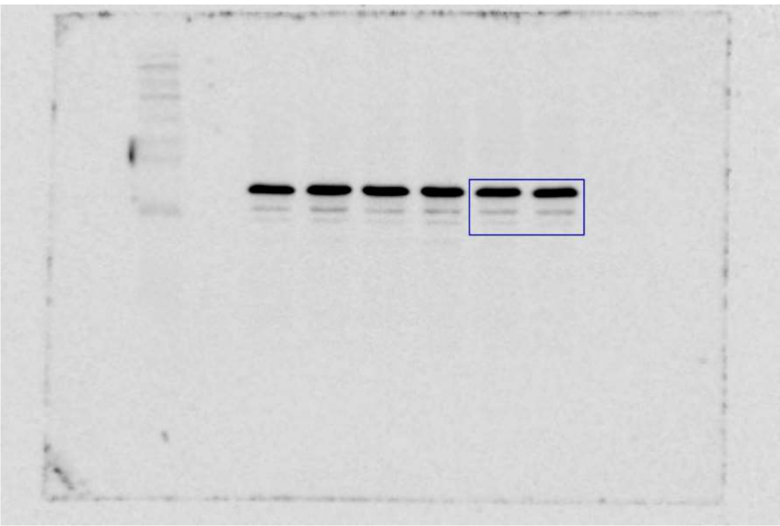

**Figure 1I**

DYRK1B

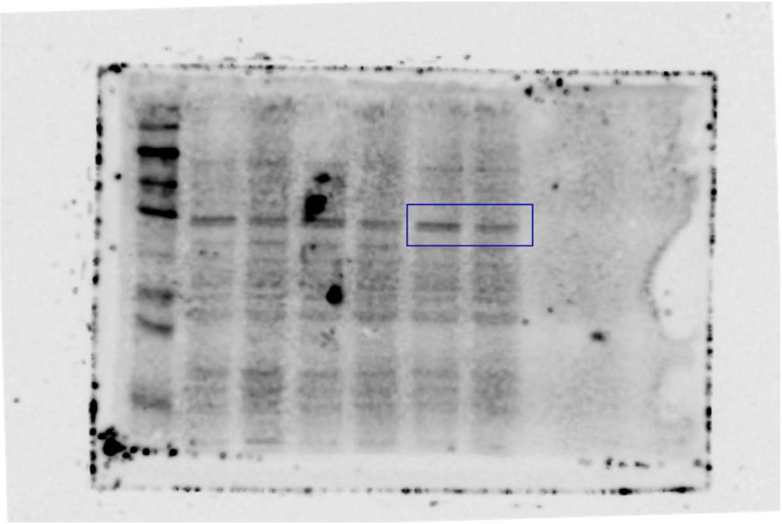

DYRK1A

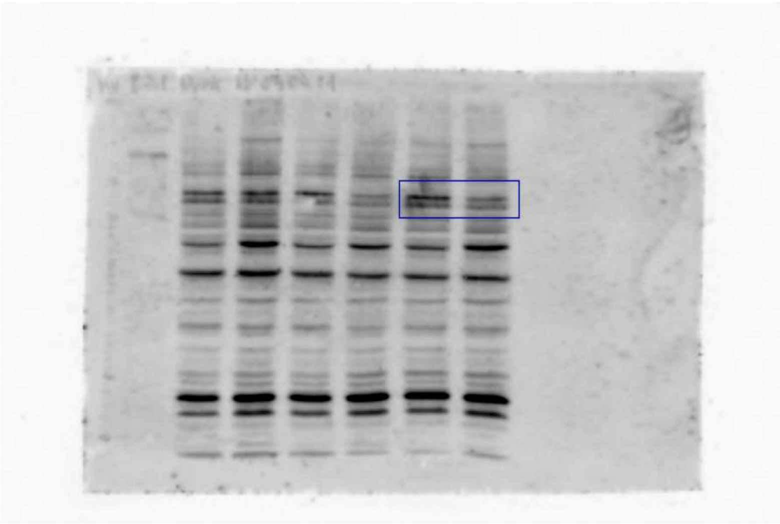

GAPDH

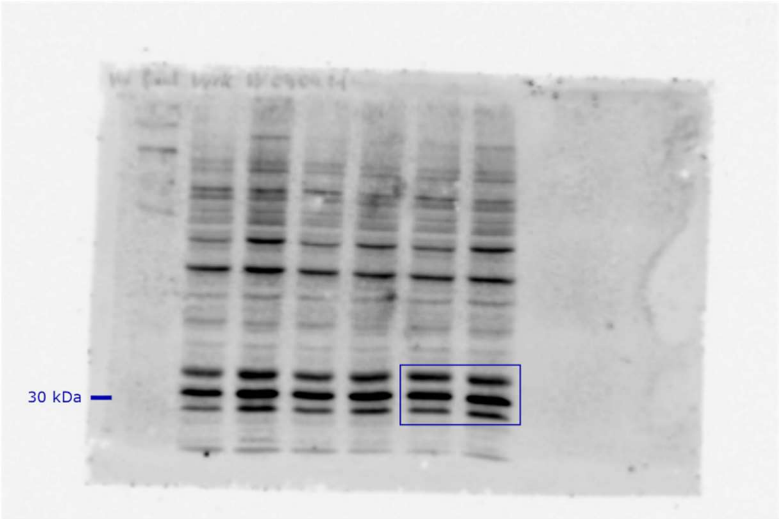


Position of the 30 kDA standard has been transferred by alignment of the GAPDH and DYKR1B blot using the GIMP software

p-4E-BP1 (Thr37/46)

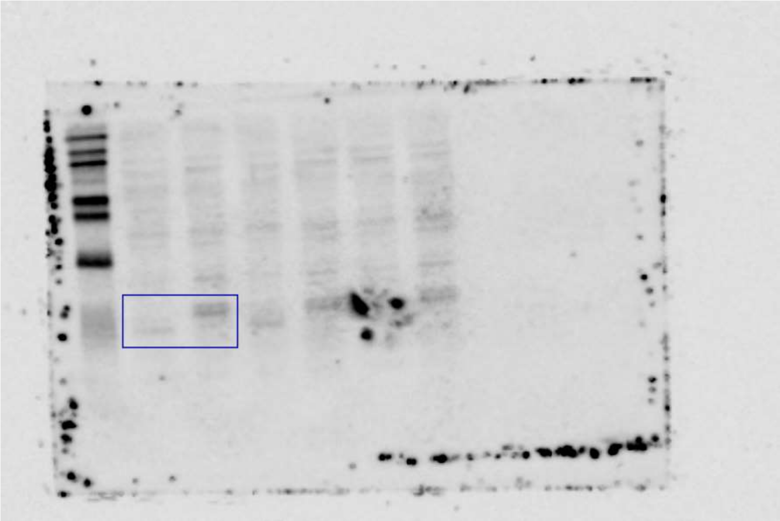

GAPDH (p-4E-BP1 – Blot)

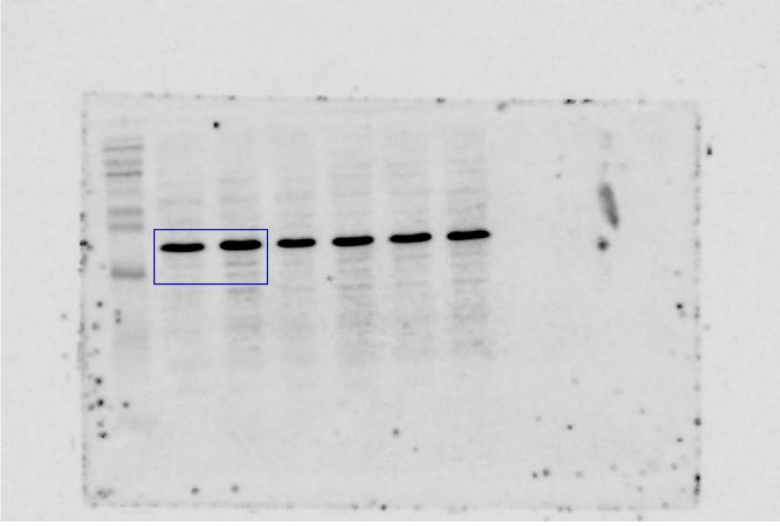

**Figure 2A**

DYRK1B

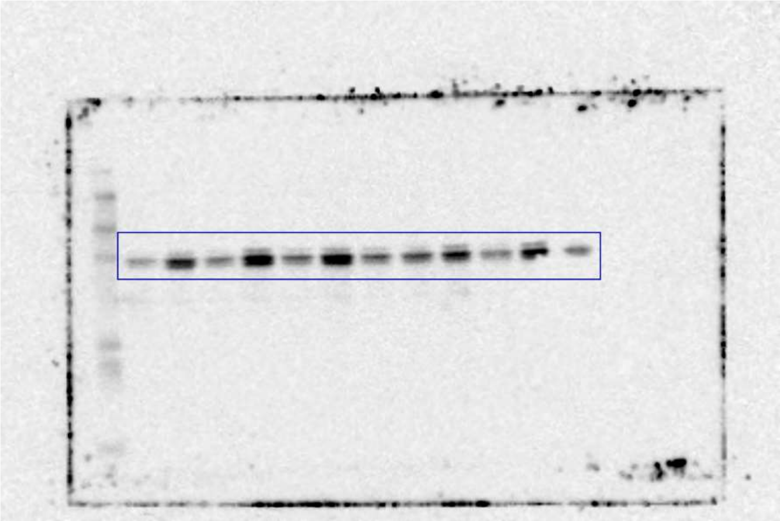

DYRK1A

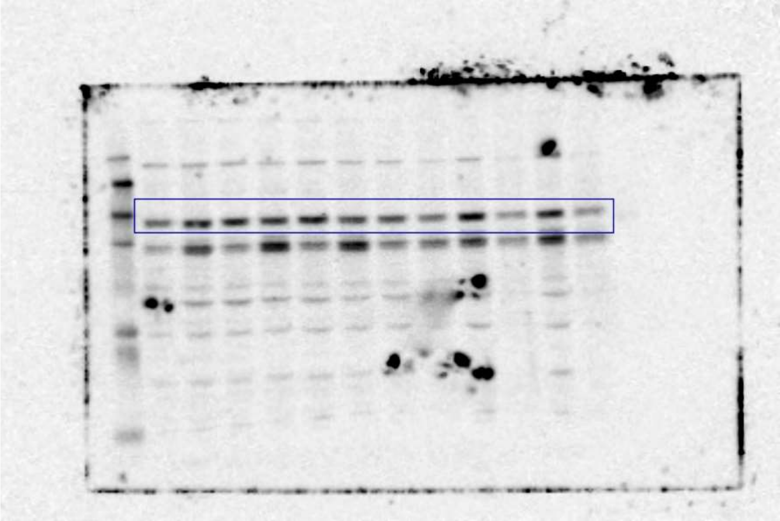

GAPDH

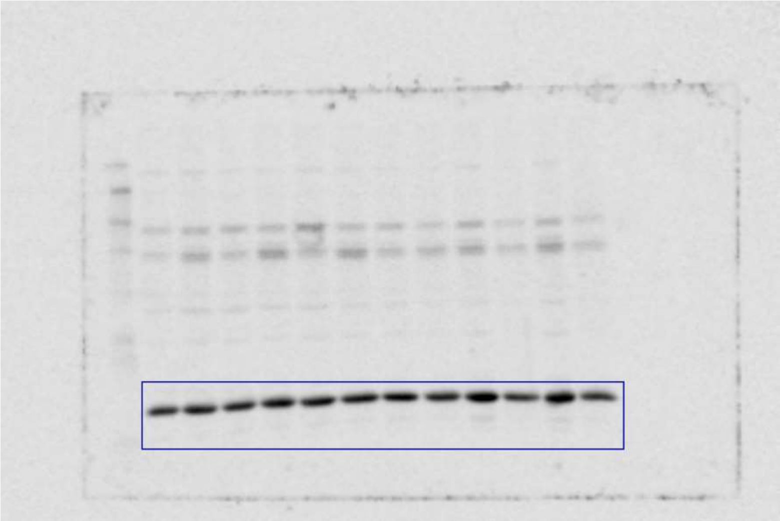

**Figure 2C**

DYRK1B

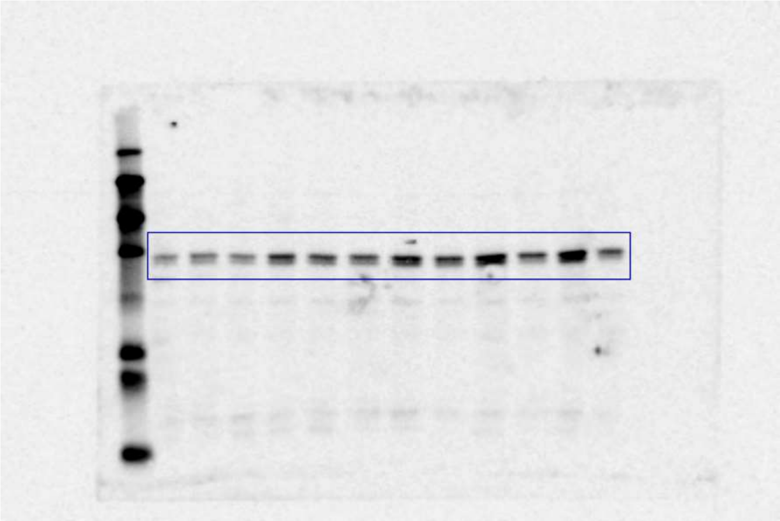

DYRK1A

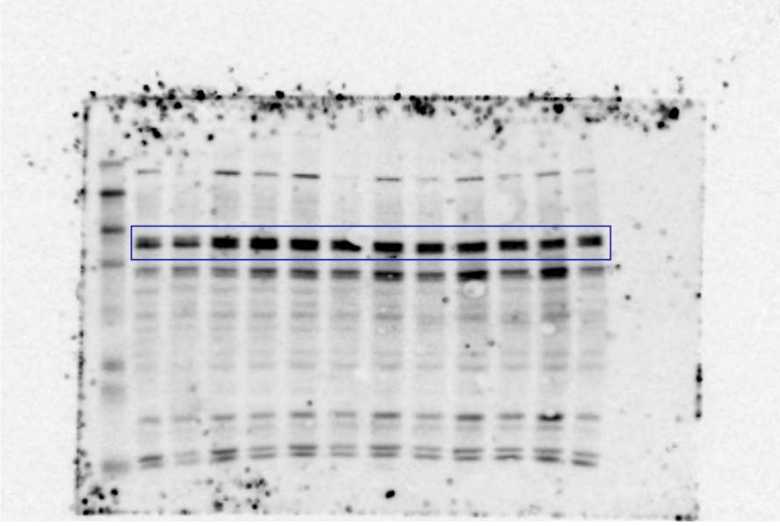

GAPDH

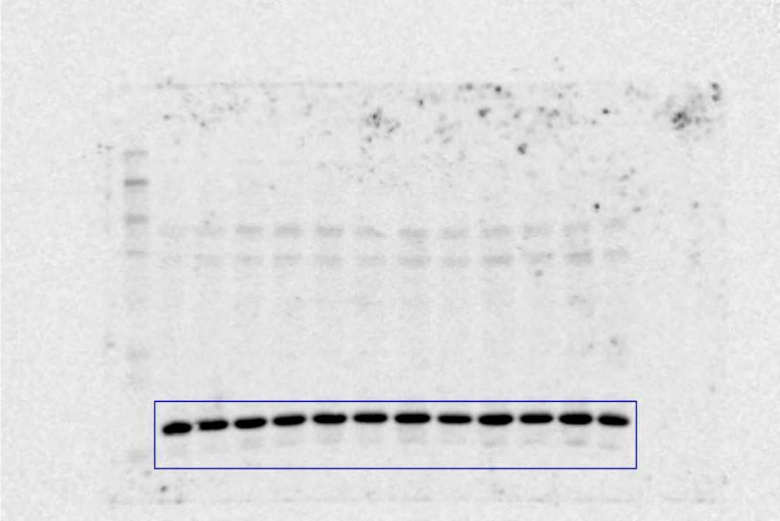

**Figure 3A**

DYRK1B

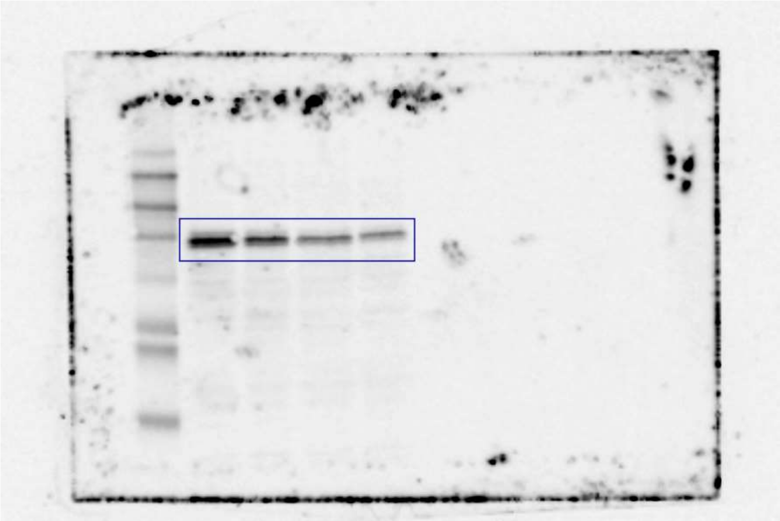

DYRK1A

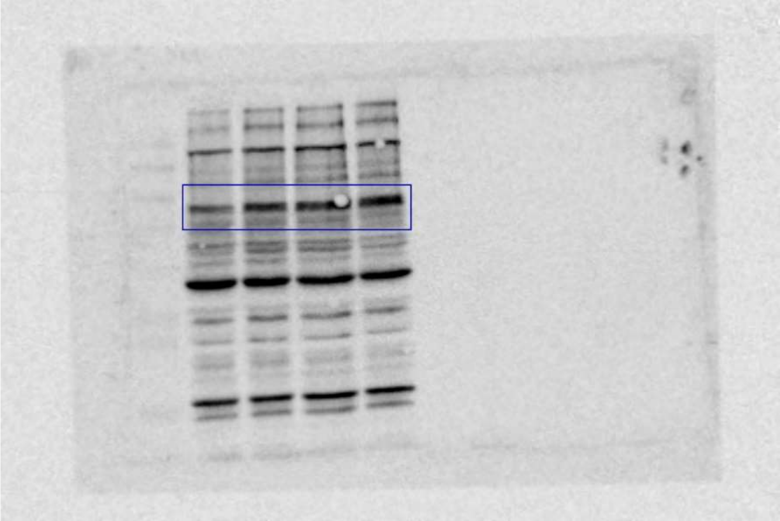

GAPDH

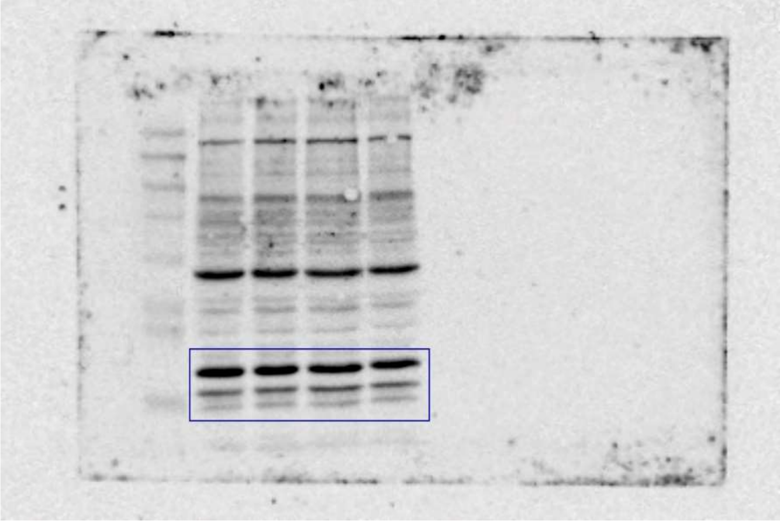

YAP

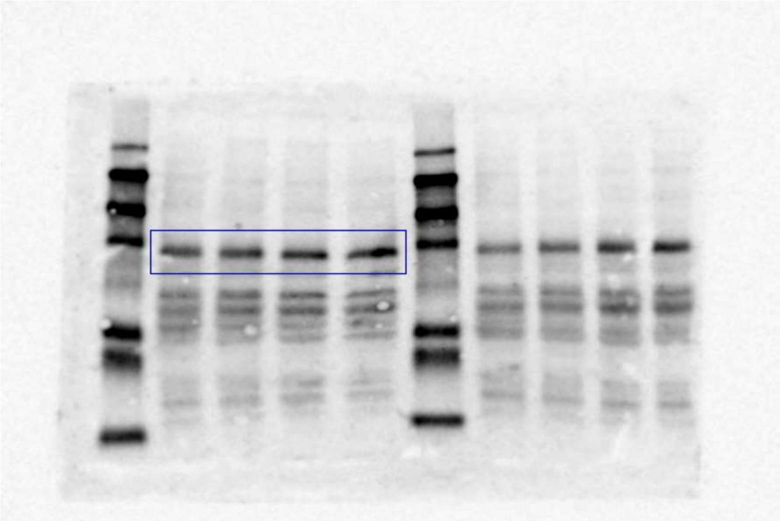

GAPDH (YAP-Blot)

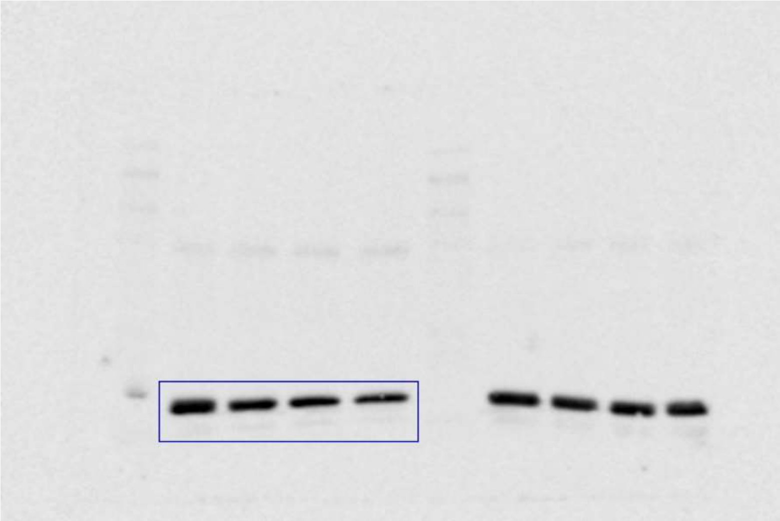

**Figure 3C**

DYRK1B

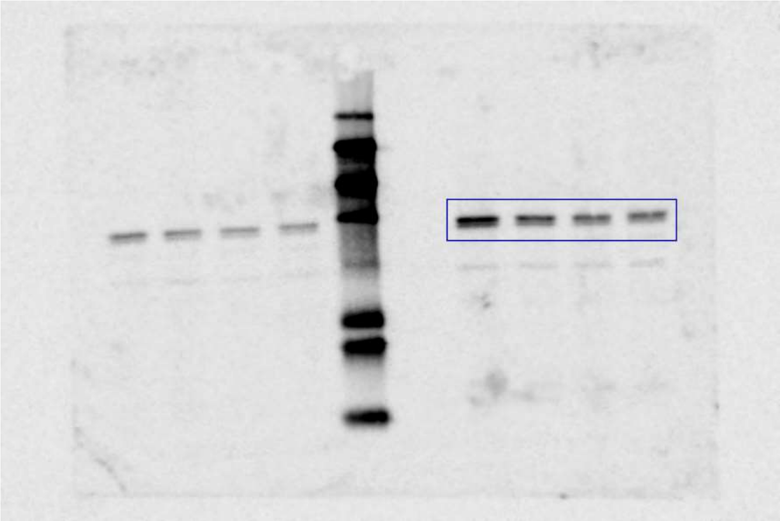

DYRK1A

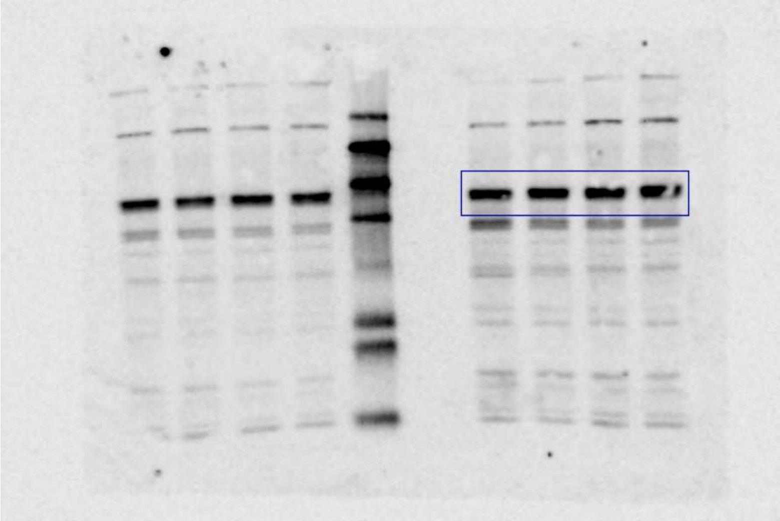

GAPDH

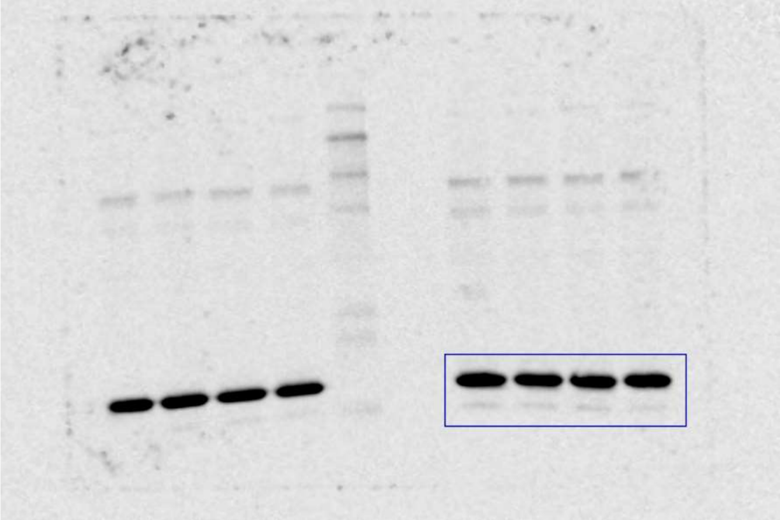

YAP

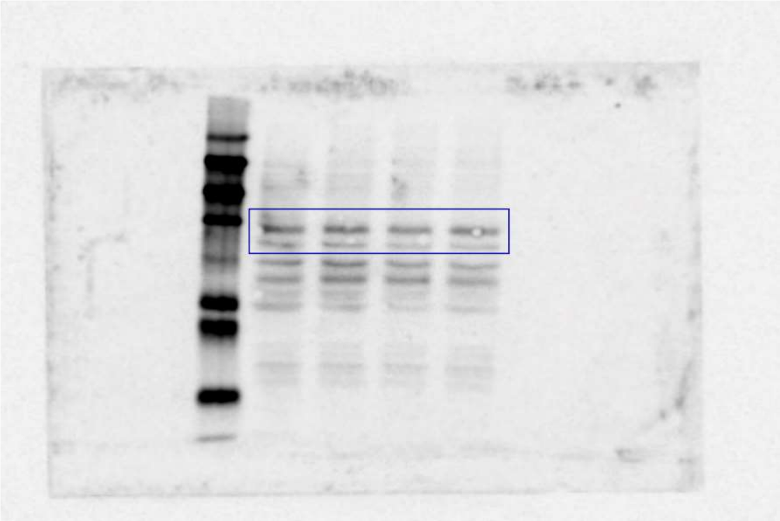

GAPDH (YAP)

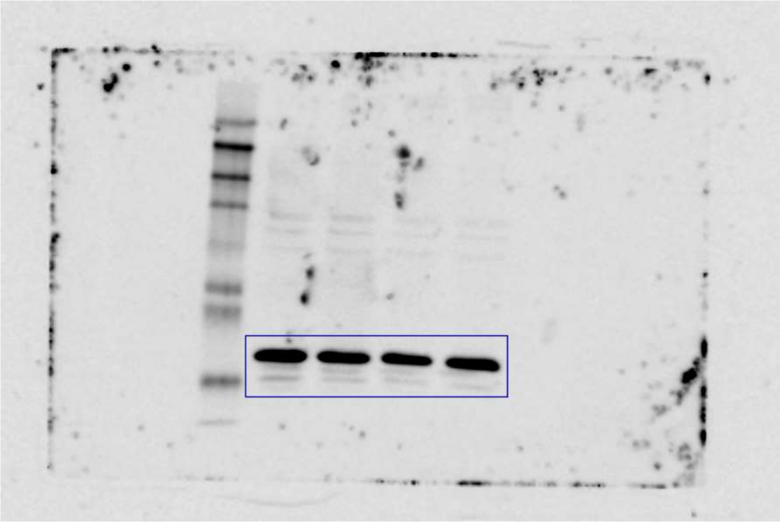

**Figure 4B**

DYRK1B

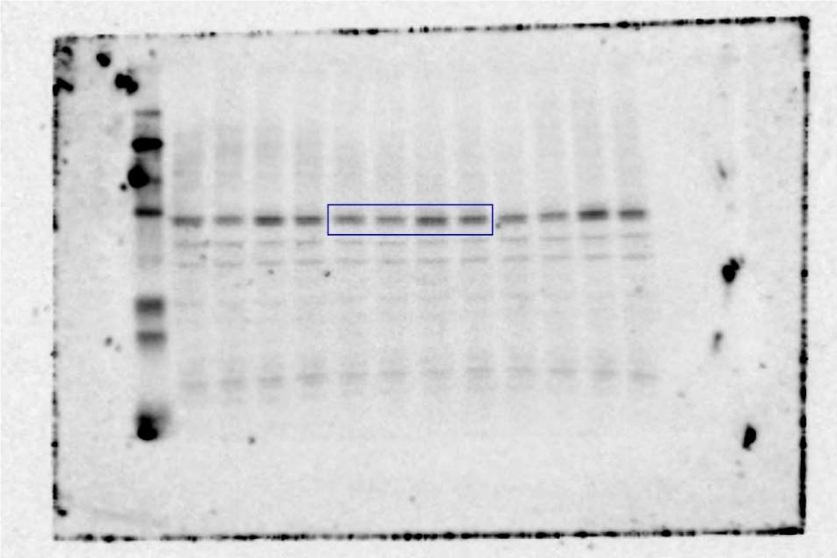

DYRK1A

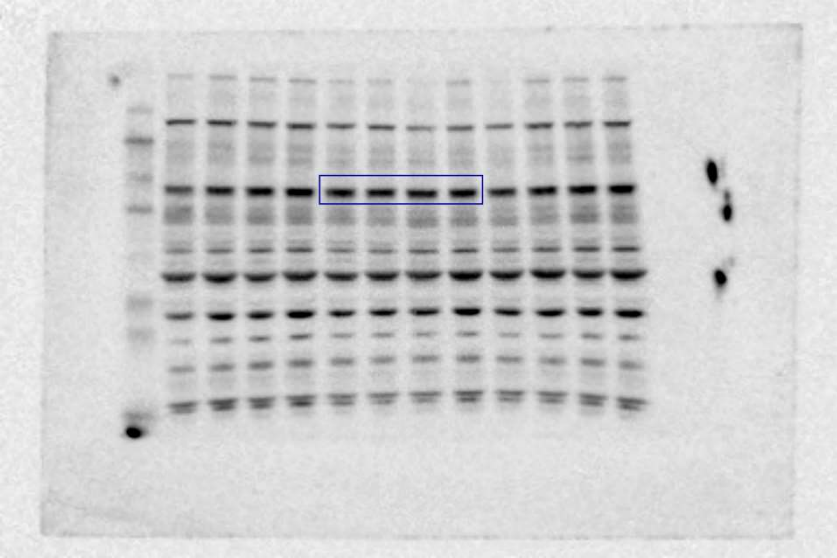

GAPDH

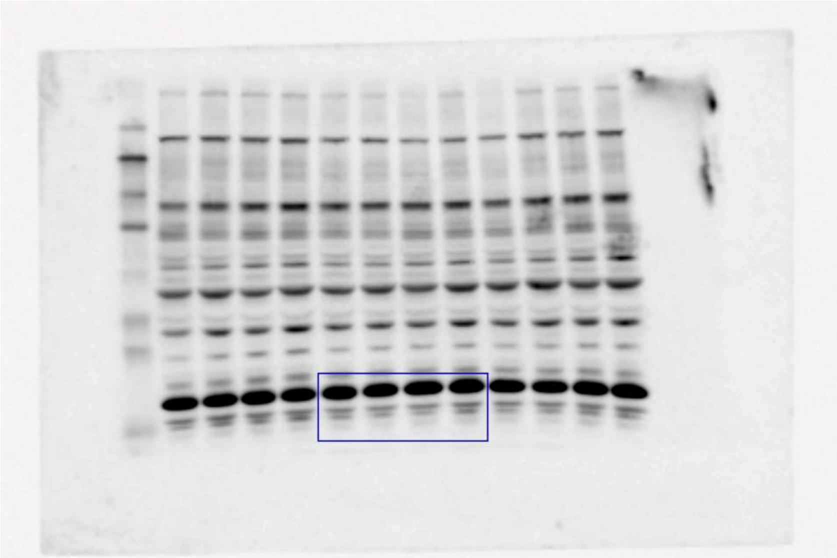

p-YAP (Ser127)

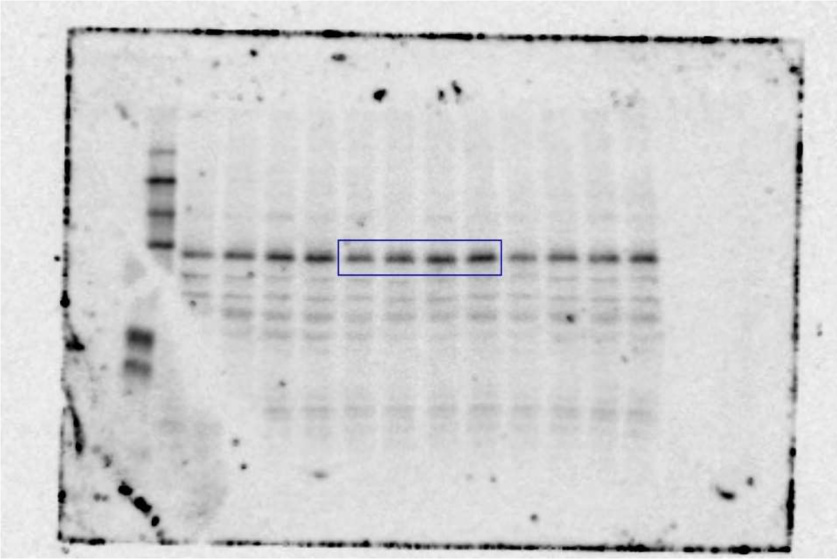

GAPDH (YAP-Blot)

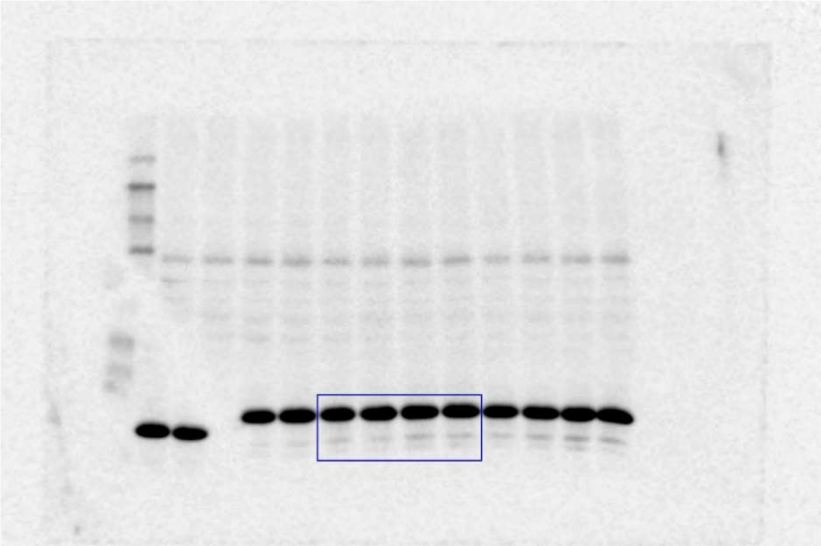

**Figure 4C**

DYRK1B

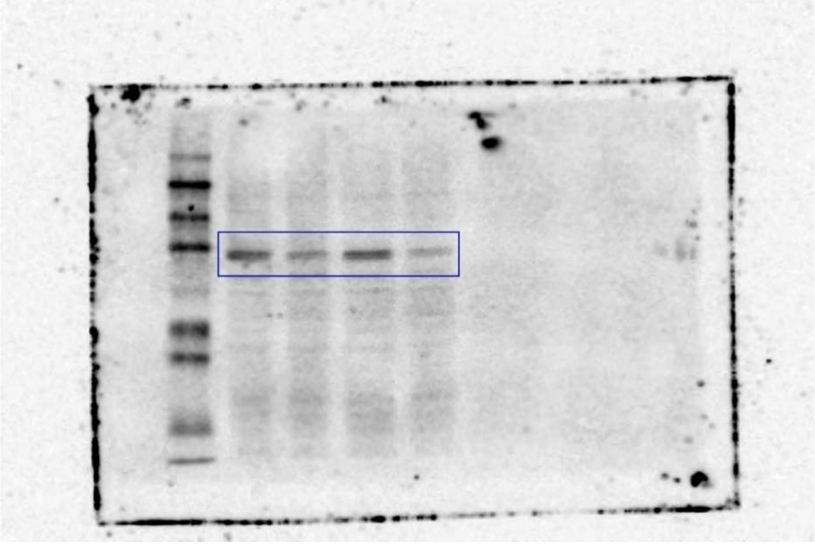

DYRK1A

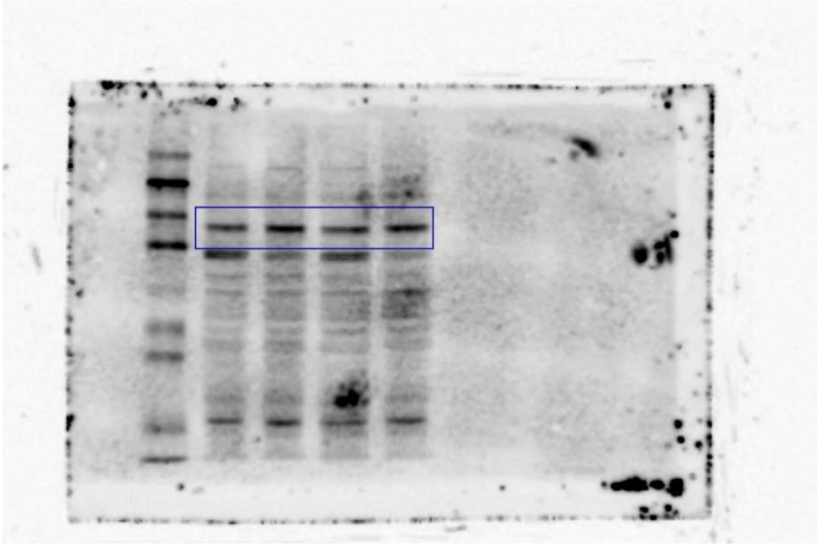

GAPDH

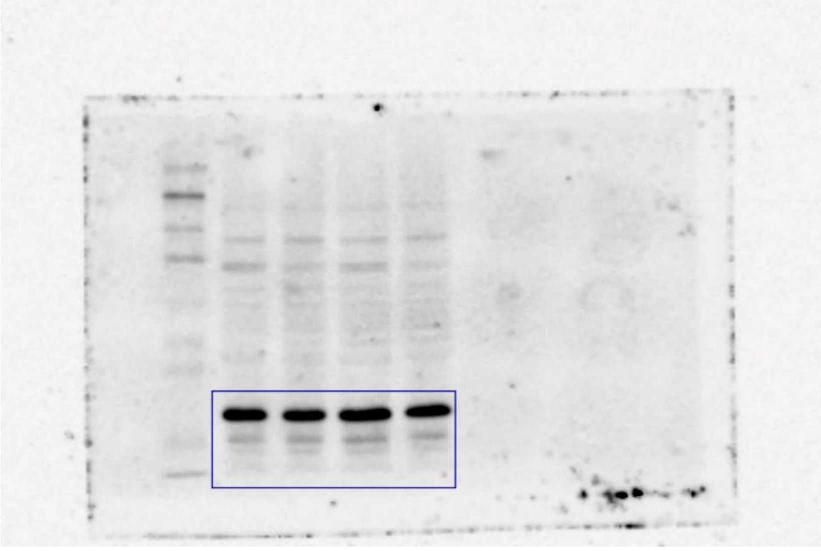

p-YAP (Ser127)

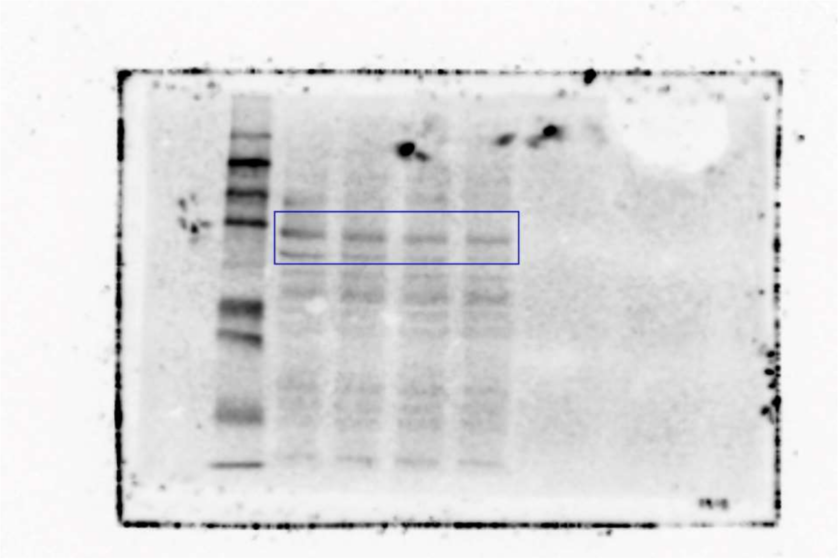

GAPDH (YAP-Blot)

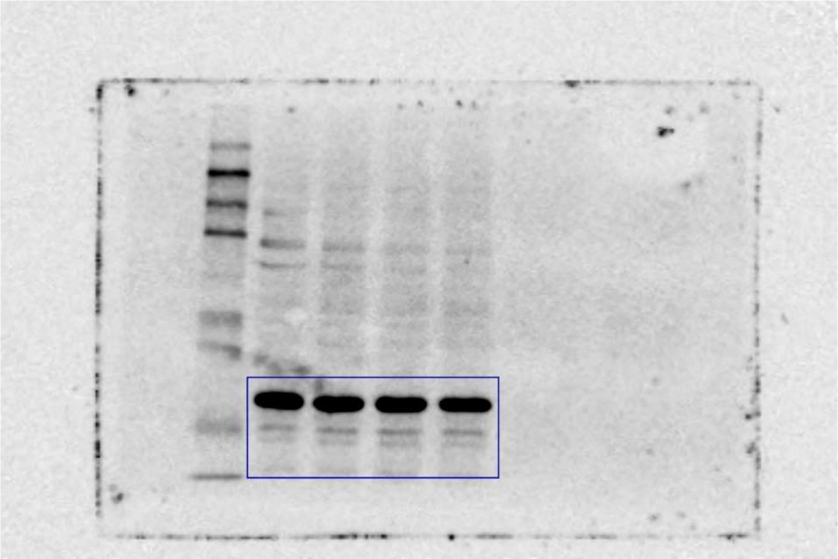

**Figure 5B**

DYRK1B

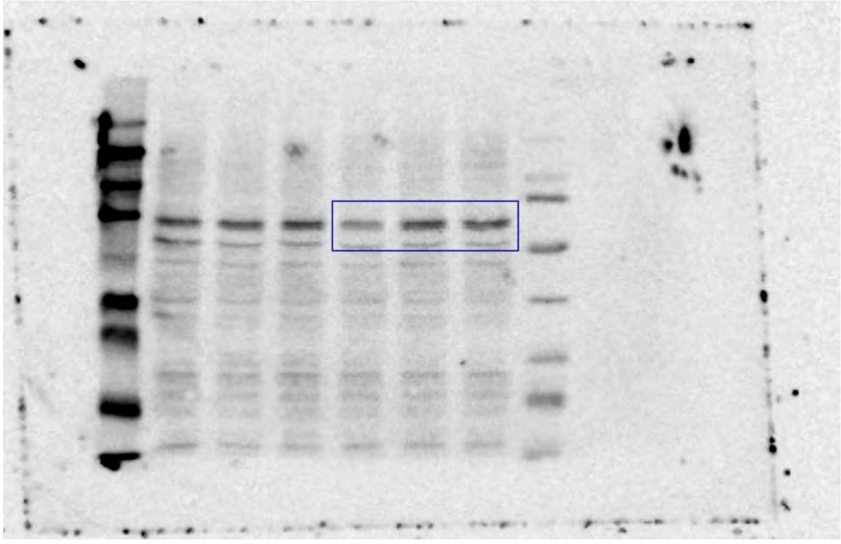

DYRK1A

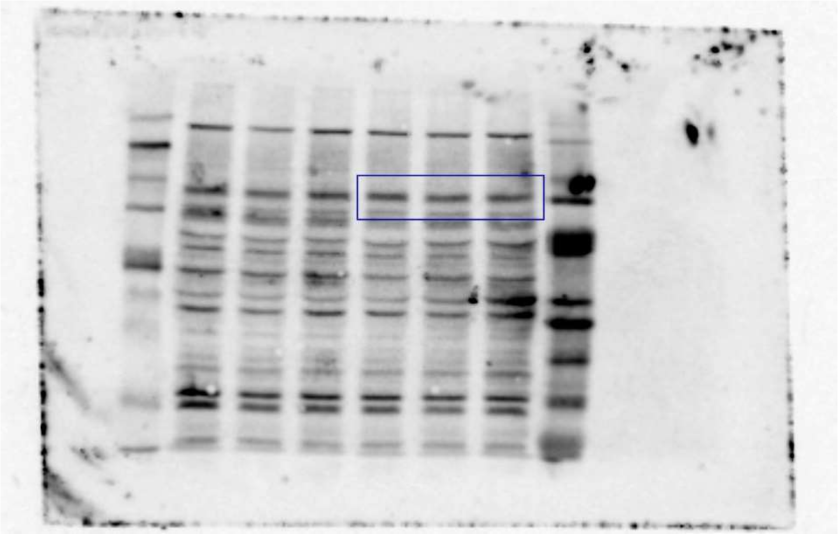

GAPDH

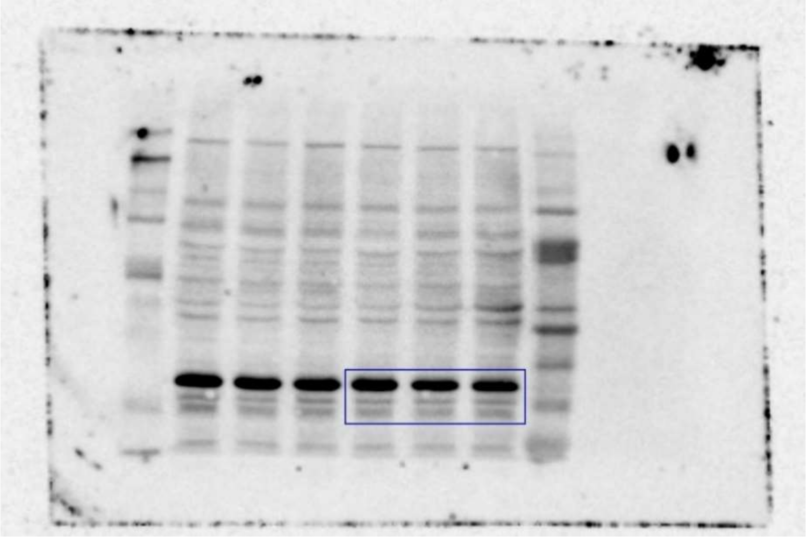

p-H3

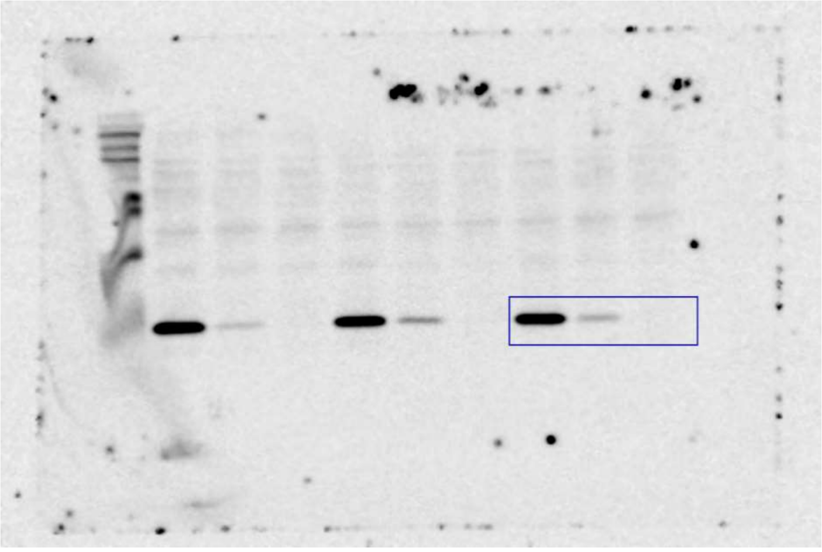

GAPDH (p-H3)

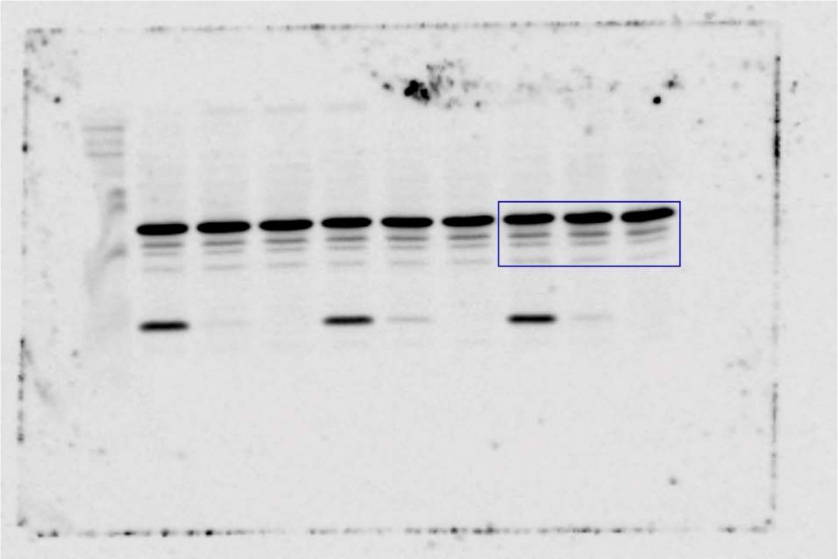

**Figure 5D**

DYRK1B

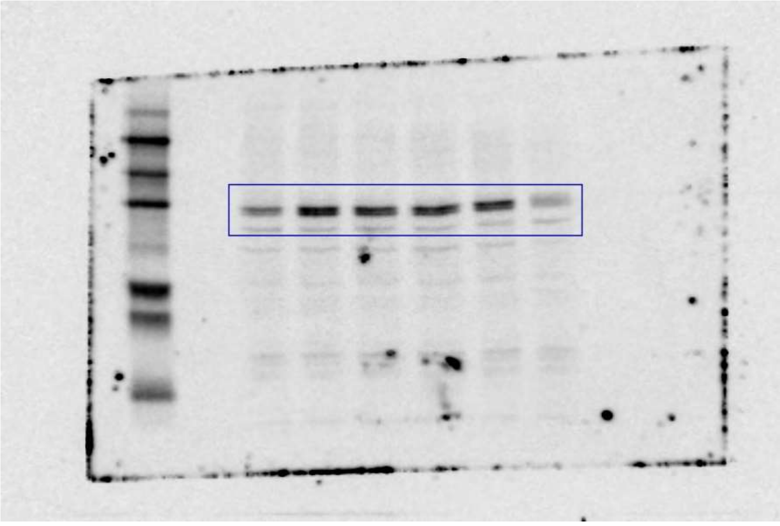

p-Aurora A (Thr288) / Aurora B (Thr232) / Aurora C (Thr198)

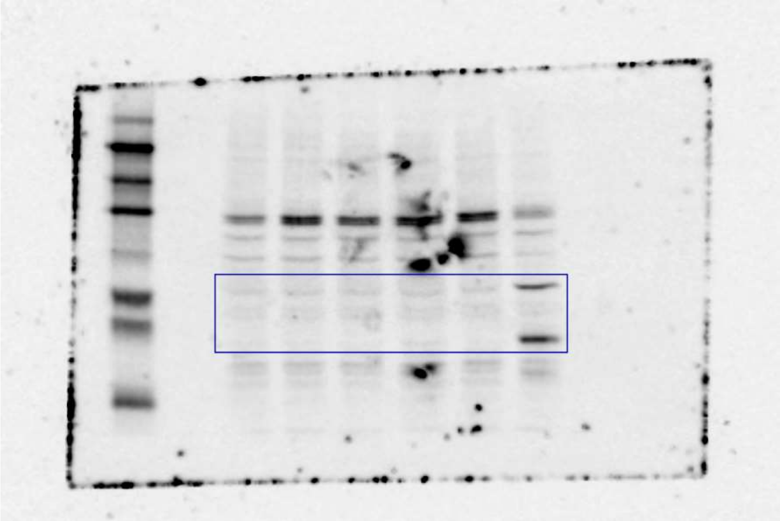

GAPDH

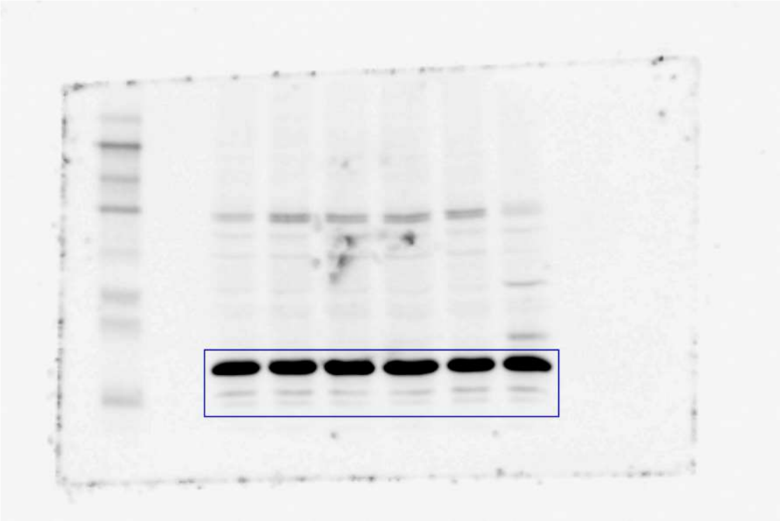

**Figure S3A**

DYRK1B

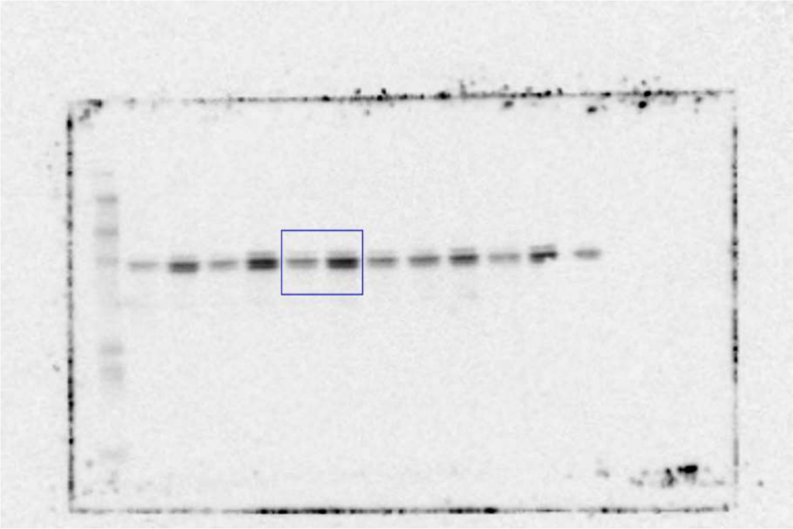

Blot is identical to Figure 2A

**Figure S3B**

DYRK1B

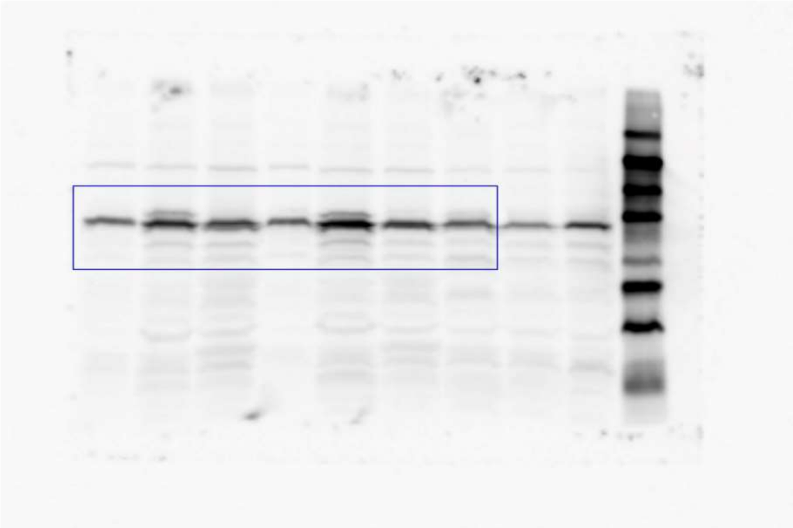

GAPDH

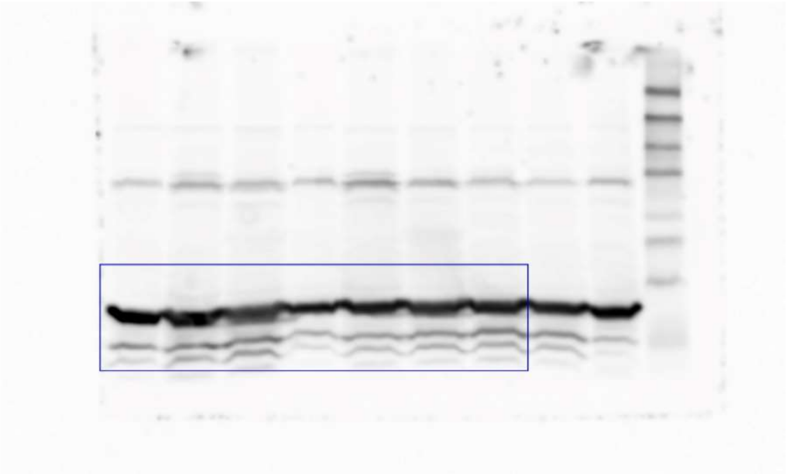

**Figure S6A**

DYRK1B

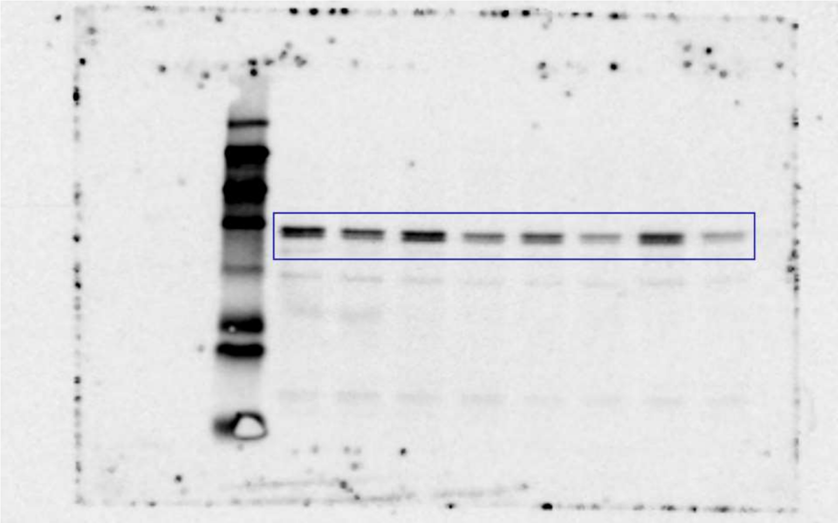

DYRK1A

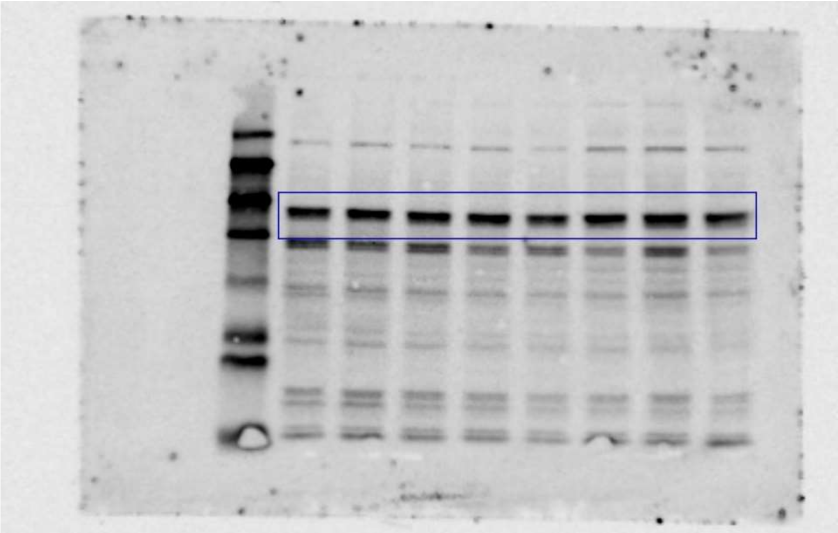

GAPDH

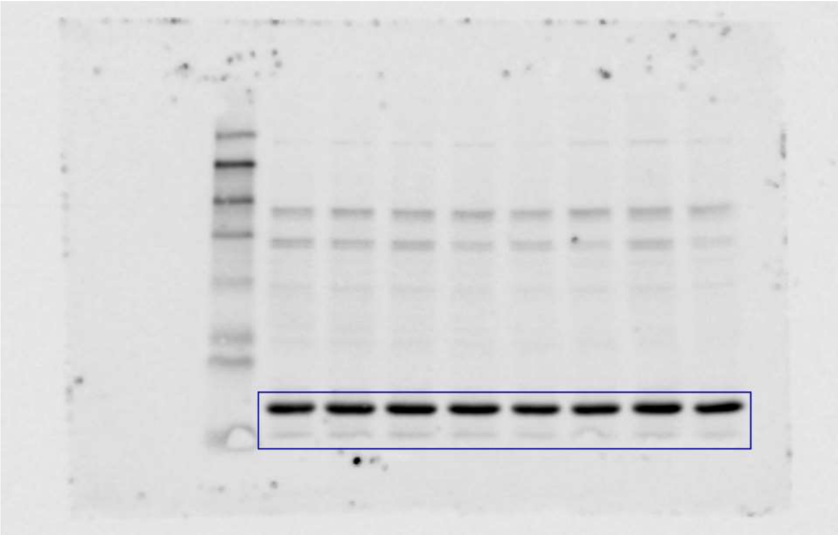

**Figure S6B**

DYRK1B

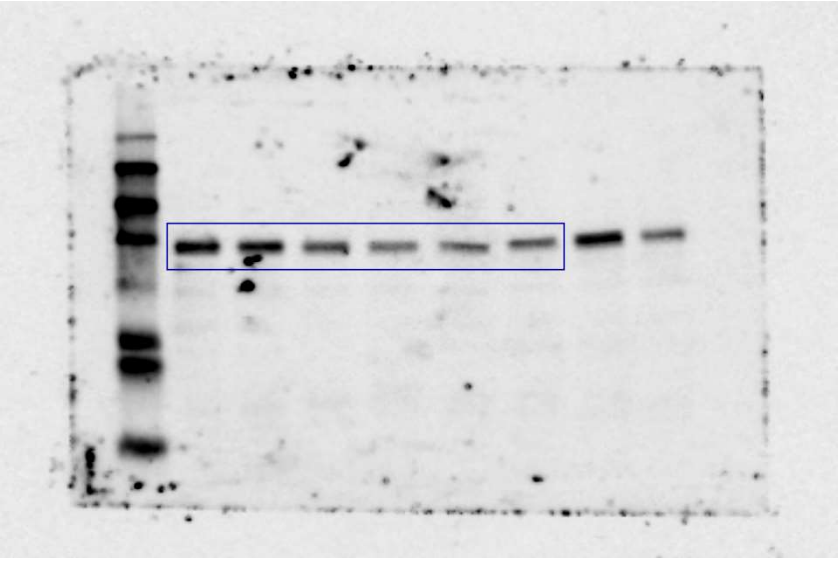

DYRK1A

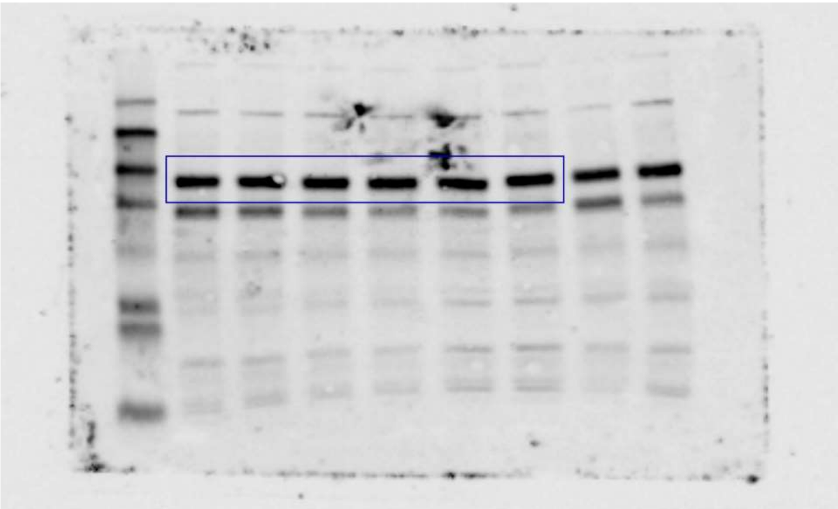

GAPDH

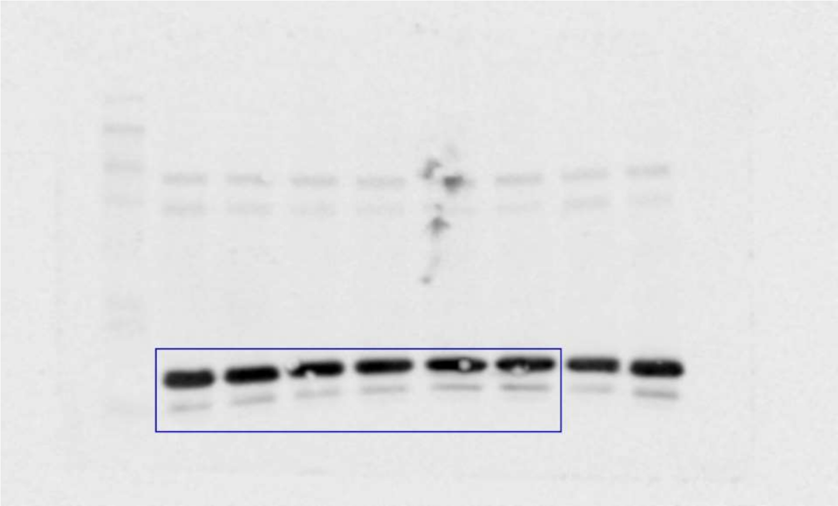

**Figure S6C**

DYRK1B

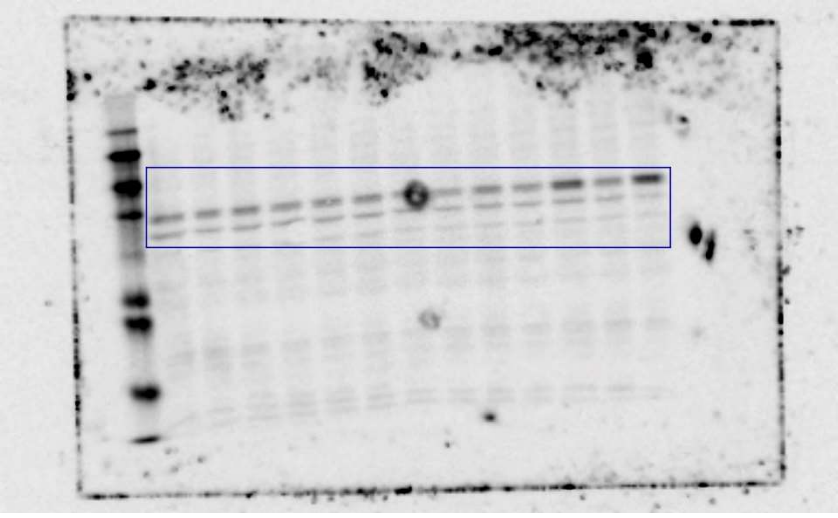

DYRK1A

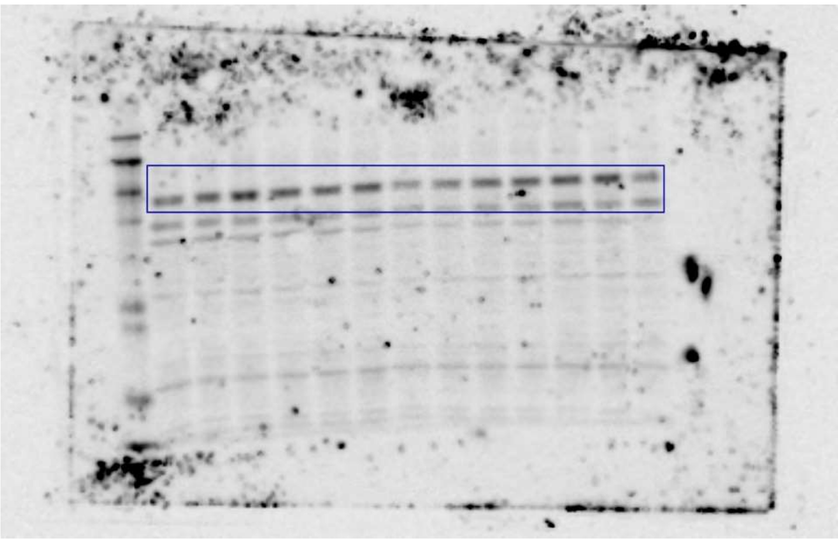

GAPDH

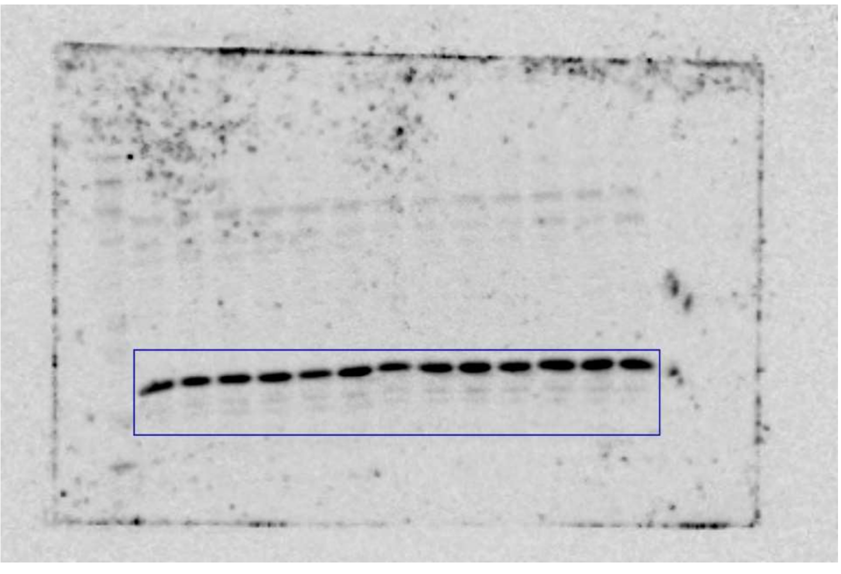

**Figure S7A**

p-AURKA(Thr288) / p-AURKB(Thr232)

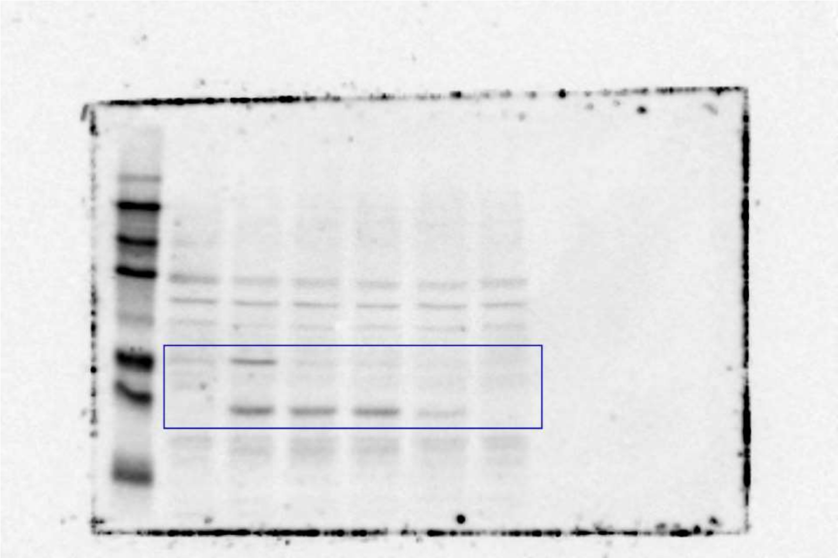

GAPDH

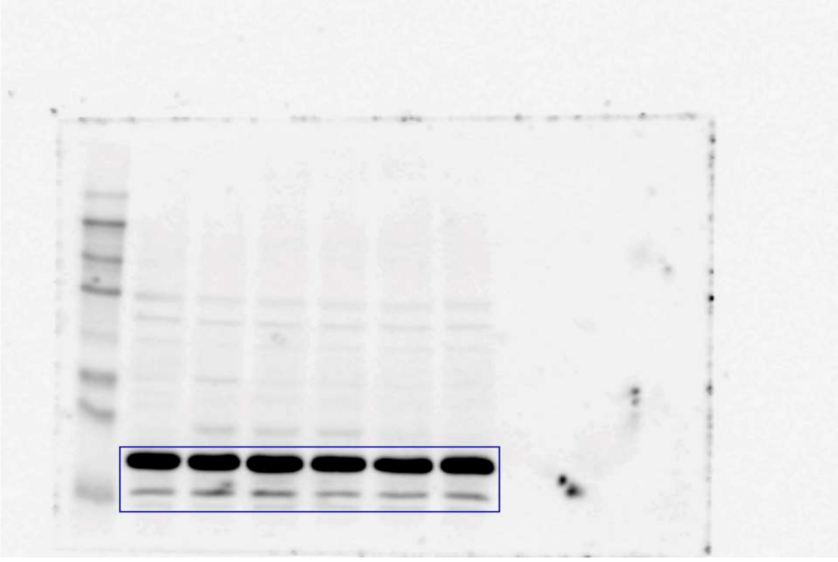

**Figure S7B**

DYRK1B

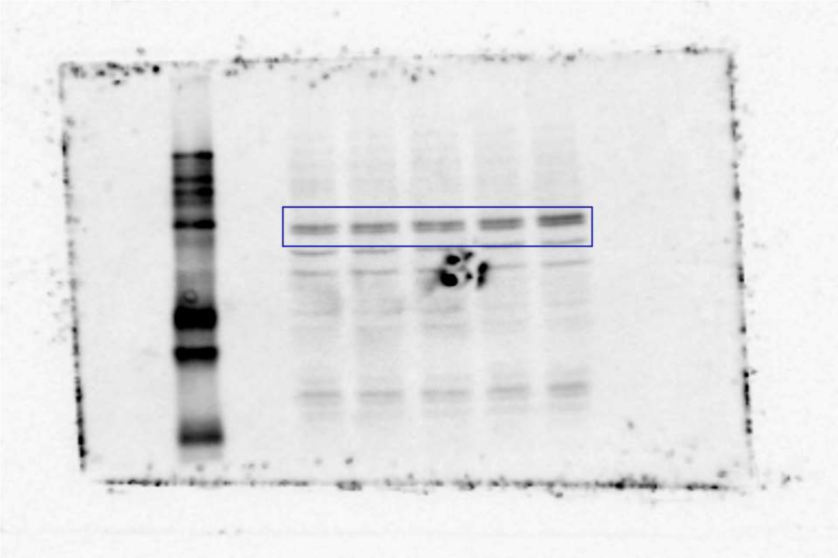

GAPDH

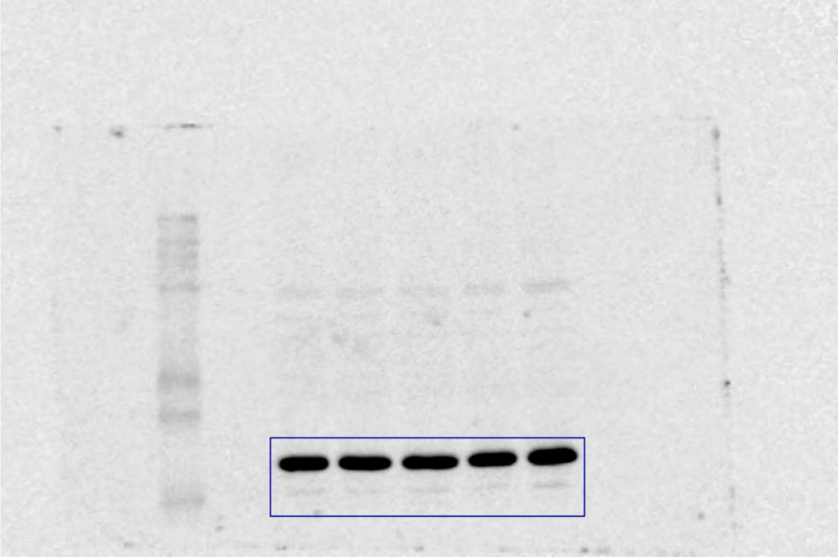

Supplement: Supplementary file 1 — Supplementary Material 1 [file 41598_2024_74190_MOESM1_ESM.pdf]
